# Understanding High-Salt and Cold Adaptation of a Polyextremophilic Enzyme

**DOI:** 10.3390/microorganisms8101594

**Published:** 2020-10-16

**Authors:** Ram Karan, Sam Mathew, Reyhan Muhammad, Didier B. Bautista, Malvina Vogler, Jorg Eppinger, Romina Oliva, Luigi Cavallo, Stefan T. Arold, Magnus Rueping

**Affiliations:** 1 KAUST Catalysis Center, King Abdullah University of Science and Technology (KAUST), Thuwal 23955-6900, Saudi Arabia; sam.mathew@kaust.edu.sa (S.M.); didier.barradasbautista@kaust.edu.sa (D.B.B.); Malvina.Vogler@kaust.edu.sa (M.V.); jorg.eppinger@gmail.com (J.E.); oliva@uniparthenope.it (R.O.); luigi.cavallo@kaust.edu.sa (L.C.); 2 Computational Bioscience Research Center (CBRC), King Abdullah University of Science and Technology (KAUST), Biological and Environmental Science and Engineering (BESE), Thuwal 23955-6900, Saudi Arabia; reyhan.muhammad@kaust.edu.sa; 3 Department of Sciences and Technologies, University Parthenope of Naples, Centro Direzionale Isola C4, I-80143 Naples, Italy; 4 Centre de Biochimie Structurale, CNRS, INSERM, Université de Montpellier, 34090 Montpellier, France

**Keywords:** extremophiles, halophiles, psychrophiles, polyextremophiles, extremozymes, X-ray crystallography, molecular dynamics simulations

## Abstract

The haloarchaeon *Halorubrum lacusprofundi* is among the few polyextremophilic organisms capable of surviving in one of the most extreme aquatic environments on Earth, the Deep Lake of Antarctica (−18 °C to +11.5 °C and 21–28%, w/v salt content). Hence, *H. lacusprofundi* has been proposed as a model for biotechnology and astrobiology to investigate potential life beyond Earth. To understand the mechanisms that allow proteins to adapt to both salinity and cold, we structurally (including X-ray crystallography and molecular dynamics simulations) and functionally characterized the β-galactosidase from *H. lacusprofundi* (hla_bga). Recombinant hla_bga (produced in *Haloferax volcanii*) revealed exceptional stability, tolerating up to 4 M NaCl and up to 20% (v/v) of organic solvents. Despite being cold-adapted, hla_bga was also stable up to 60 °C. Structural analysis showed that hla_bga combined increased surface acidity (associated with halophily) with increased structural flexibility, fine-tuned on a residue level, for sustaining activity at low temperatures. The resulting blend enhanced structural flexibility at low temperatures but also limited protein movements at higher temperatures relative to mesophilic homologs. Collectively, these observations help in understanding the molecular basis of a dual psychrophilic and halophilic adaptation and suggest that such enzymes may be intrinsically stable and functional over an exceptionally large temperature range.

## 1. Introduction

Enzymes have emerged as preferred tools in green chemistry and have the potential to play a vital role in sustainable development in chemical, biotechnological, bioremediation, agricultural and pharmaceutical industries [1,2]. Over the decades, thousands of enzymes with remarkable catalytic abilities have been discovered, and the use of bioengineered enzymes as industrial catalysts has continuously increased in recent years [3]. However, the total number of industrial applications of these enzymes remains rather modest, mainly due to the absence of good operational stability in the presence of high salt and/or organic solvents, and insufficient catalytic activity at high or low temperatures [2,4,5]. Although improvements were achieved by protein engineering, these procedures are often lengthy and expensive with non-generalizable outcomes, because increased enzyme stability mostly results from specific mutations, which usually do not obey any obvious trends or patterns [6,7,8,9,10,11]. Alternatively, nature provides enzymes from extremophilic microorganisms that have a unique ability to grow and thrive in extreme environments such as volcanic areas, hypersaline lakes, alkaline soda lakes, deserts, and cold oceans [7,12,13,14,15,16,17,18]. Since the metabolic processes and physiological functions of extremophiles are adapted to prevail under harsh conditions, enzymes from these microorganisms, called extremozymes, possess unique features enabling them to carry out reactions under extreme conditions, such as the presence of up to 5.2 M salt, various surfactants, organic solvents, elevated or low temperature, and at alkaline pH [14,19]. Conversely, enzymes from mesophilic sources function less efficiently or become denatured under such conditions [14,20].

Salt and cold-active enzymes are particularly attractive for biotechnology. The high stability of enzymes towards salt entails tolerance to low water activity, such as prevailing in mixtures of aqueous and organic or non-aqueous media and low temperatures can save energy and eliminate microbial contamination [14]. These characteristics increase the enzymes’ potential as industrial biocatalysts because organic solvents are often used to improve the solubility of hydrophobic substrates, alter the hydrolytic as well as the kinetic equilibrium and therefore have the potential to increase the yield and specificity of the product [15]. Cold-active enzymes have numerous applications in food processing, as a detergent additive, biotransformation and bioremediation in cold climates and low-energy wastewater treatment [9,10,21,22,23,24].

Beyond biotechnology, salt and cold-active proteins are also of high scientific interest. High surrounding salt concentrations cause exo-osmosis, pulling out cytoplasmic water, and dehydrating the cell [14,25]. Cold temperatures critically affect the hydration sphere of a protein and restrict its conformational mobility required for efficient catalysis [22,26]. Hence, the adaptation of enzymes to both cold and salinity has attracted much interest. Stably folded extremophilic proteins may have formed during prebiotic ages, suggesting a critical role of these proteins in the origin of life [27]. Studies of extremophilic proteins may not only help to understand the early evolution of life on Earth, but also provide clues for how life could potentially survive on other planets [28,29,30,31].

Despite their industrial and biological interest, relatively little is known about the structure and function of poly-extremozymes. *Halorubrum lacusprofundi*, a completely sequenced extremely halophilic archaeon isolated from Deep Lake Antarctica is an attractive system for detailed structural and functional analysis of protein adaptation in extreme conditions and of special interest for astrobiology [28,30]. Antarctic Deep Lake is one of the coldest and most extreme aquatic environments on earth. This lake has never been reported to freeze, even at temperatures below −20 °C because of its extreme salinity (28% w/v). Due to its extreme conditions, its microbial diversity is very low [32]. The Antarctic Deep Lake has long been of great interest for marine biologists and astrobiologists as an environmental analog for icy moons and planets, such as Jupiter’s moon Europa. Very recently, multiple subglacial water bodies were detected in the south pole of Mars. Interestingly, the researchers suggested that these water bodies were hypersaline perchlorate brines similar to the subglacial lakes found in Antarctica [33].

Here, we report the biochemical and structural characterization of the β-galactosidase enzyme from *H. lacusprofundi* (hla_bga), which belongs to glycoside hydrolase family 42 and exhibits psychrophilic and halophilic properties. β-galactosidases (E.C. 3.2.1.23) catalyze the hydrolysis of lactose into glucose and galactose, as well as the transgalactosylation of prebiotic galacto-oligosaccharides. In Antarctic environments where lactose is not available, hla_bga may most likely function in breaking down short-chain oligosaccharides released from pectin galactans. We demonstrate that hla_bga combines features enabling halophily with a fine-tuned protein flexibility to enhance catalysis at low temperatures. Thus, our study provides rationales for the biological and industrial enzyme adaptation to polyextreme conditions.

## 2. Materials and Methods

### 2.1. Chemicals and Reagents

Restriction enzymes, Q5 DNA polymerase and a Gibson Assembly Cloning Kit were purchased from New England Biolabs (Ipswich, MA, USA). O-nitrophenyl-β-d-galactopyranoside (ONPG) were purchased from ThermoFisher Scientific (Waltham, MA, USA). All other chemicals were purchased from Sigma-Aldrich (St. Louis, MO, USA). Water used was desalted and purified using a milli-Q^®^ (Merck, Darmstadt, Germany) system.

### 2.2. Strains, Plasmids, Media, and Culture Conditions

*Halorubrum lacusprofundi* isolated from Deep Lake Antarctica [34] was obtained from the German Collection of Microorganisms and Cell Cultures (DSM No.: 5036). It was grown in Deutsche Sammlung von Mikroorganismen und Zellkulturen (DSMZ) medium 372 at 30 °C with shaking. *Escherichia coli* One Shot^®^ TOP10 chemical competent cells were purchased from Invitrogen (Karlsbad, CA, USA). *Escherichia coli* derivatives harboring cloning plasmid used in this study were grown at 37 °C in Luria–Bertani (LB) medium supplemented with 100 μg/mL ampicillin. *Haloferax volcanii* H1895 and its corresponding vector pTA96346 were kindly provided by Dr. Thorsten Allers (Institute of Genetics, School of Biology, University of Nottingham, Queen’s Medical Centre, Nottingham, UK). *Haloferax volcanii* and derivatives were cultured in the Hv-YPC medium at 45 °C with shaking as previously described [15,35]. For solid media, 2% (w/v) agar was added, and when required, 5-bromo-4-chloro-indolyl-β-d-galactopyranoside (X-Gal) was added to 40 μg/mL. Stock cultures were maintained in glycerol at −80 °C. For short-term use, purified cultures were maintained on stock plates at 4 °C.

### 2.3. Expression of the β-Galactosidase Gene in Haloferax Volcanii

The β-galactosidase (*bga*) gene from *H. lacusprofundi* was PCR amplified from the genome and cloned via NdeI and BamHI restriction enzymes using the Gibson Assembly Cloning Kit into pTA963, to generate the pTA963_bga expression plasmid (Figure S1). The construct w2018as validated by restriction digestion using NdeI and BamHI, PCR amplification, and DNA sequencing. Primers used for amplification and sequencing are listed in Table S1. β-galactosidase gene containing vector, pTA963_bga was transformed into the *Haloferax volcanii* H1895 using the polyethylene glycol/ethylenediaminetetraacetic acid(PEG/EDTA) method. Transformants were screened on Hv-YPC medium plates containing 40 μg/mL X-Gal [35,36] (Figure S2). Expression was carried out as described earlier [15,35].

### 2.4. β-Galactosidase Purification

Cells were harvested by centrifugation (6000× *g*, 4 °C, 10 min) in a 5430R centrifuge (Eppendorf, Germany) and disrupted in binding buffer (20 mM 4-(2-hydroxyethyl)-1-piperazineethanesulfonic acid (HEPES) buffer pH 7.4 containing 2.0 M NaCl, 10% v/v glycerol and 30 mM imidazole) containing cOmplete protease inhibitor cocktail (Roche, Indianapolis, IN, USA) using a sonicator (Model Q500, QSONICA, Newtown, CT, USA) with a 1.9 cm probe (Thermo Scientific, Waltham, USA). Cell debris was removed by centrifugation (25,000× *g*, 4 °C, 10 min) in an Avanti J-26 XP centrifuge (Beckman Coulter) and resulting crude extract was filtered through a Nalgene membrane filter (pore size, 0.2 μm). The supernatant was loaded at a flow rate of 1.0 mL/min onto a 5-mL HiTrap Ni^2+^ chelating column (GE Healthcare Life Sciences, Piscataway, NJ, USA) pre-equilibrated with binding buffer. The column was washed with binding buffer, and the protein was eluted by increasing the concentration of imidazole (30 to 300 mM) in binding buffer. Aliquots were analyzed by SDS-PAGE and InVision His-Tag In-Gel Stain (Thermo Fisher Scientific, Waltham, MA, USA). The purified active fractions were combined and further purified and concentrated with Amicon^®^ Ultra-4 Centrifugal Filter Units, 30 kDa (Cat no. UFC803024). The protein was then dialyzed against 20 mM HEPES buffer pH 7.4 containing 2.0 M NaCl and 10% (v/v) glycerol. Protein concentration was determined using the absorption at 280 nm with a calculated extinction coefficient of 157,845 M^−1^ cm^−1^ and by the method of Bradford [37].

### 2.5. Tryptic Digest and LC-MS/MS Analysis

The identification of hla_bga was performed by LC-MS/MS analysis. The pure sample (20 ug) digested with trypsin using the filter aided sample preparation (FASP) protocol [38]. Peptides were measured using an Linear Trap Quadropole (LTQ)-Orbitrap mass spectrometer (Thermo Fisher Scientific, Waltham, MA, USA) and analyzed using MASCOT v2.3 (Matrix Sciences Ltd., London, UK). After trypsin digest, we detected 45 unique peptides, which covers 70% of the hla_bga sequence (Figure S3B).

### 2.6. MALDI-TOF

For MALDI-TOF measurements, a pure 20 µL protein sample of 2 mg mL^−1^ in H_2_O was desalted with a zip tip 0.6 µL C_4_ resin (Merck Millipore, Burlington, VT, USA) according to the manufacturer’s manual and eluted in 30% acetonitrile (ACN), 0.1% trifluoroacetic acid (TFA). The protein sample was mixed 1:1 with a saturated α-Cyano-4-hydroxycinnamic acid matrix in 30% ACN and 0.1% TFA. From the mixture 1 µL was spotted on a MALDI-target, air-dried and subjected for analysis using the standard protein mass detection method of BRUKER. Before each measurement, the mass detector was calibrated using protein calibration standard II (BRUKER, Billerica, MA, USA). In the electrospray spectrum, we detected singly charged (78.55 kDa), doubly charged (39.176 kDa) and triply charged peptides (26.052 kDa) (Figure S4).

### 2.7. β-Galactosidase Activity Assay

The β-galactosidase activity assay was performed as described previously [28]. Briefly, enzymatic activity was carried out in 1 mL semi-micro polymethyl methacrylate (PMMA) cuvettes (Sigma-Aldrich, St. Louis, MO, USA) for 10 min at 50 °C and pH 6.5 using 2.2 mM of the synthetic chromogenic substrate O-nitrophenyl-β-d-galactopyranoside (ONGP) as a substrate and stopped by the addition of Na_2_CO_3_ to 1.0 M concentration. The O-nitrophenol released from ONPG by β-galactosidase was measured at 420 nm using a spectrophotometer (Cary 60, Agilent, Santa Clara, CA, USA). One international unit (IU) of β-galactosidase activity is defined as the amount of enzyme liberating 1 μmol of O-nitrophenol per minute.

### 2.8. Enzyme Characterization

The enzyme was characterized for thermal stability (40–70 °C) and salt stability (0–4 M, NaCl/KCl). The stability of the enzyme was also investigated in the presence of various organic solvents (20%, v/v). Thermal stability at 50 °C was carried out in the absence and presence of different concentrations of salt (0–4.0 M salt). Samples were incubated in water bath at 50 °C for times up to 4 h. Samples were removed at fixed time intervals, cooled rapidly on ice, and the enzymatic activity was then measured under standard conditions. The results were transformed into residual activities with the highest activity on each measurement being set to 100%.

The thermal melting (Tm) curve was determined using Differential Static Light Scattertting (DSLS) measured by a Stargazer-2 (Harbinger Biotechnology, Toronto, Canada) with 1.73 mg/mL in 50 mM Tris-HCl pH 7.4 buffer containing different salt concentrations (0.5–4 M NaCl). The melting temperature values were plotted using GraphPad Prism (GraphPad Sofware Inc., La Jolla, CA, USA).

### 2.9. X-ray Crystallography

β-galactosidase protein solution was adjusted to 14 mg/mL in a solution containing 50 mM 18-Crown-6, 50 mM Tris-HCl pH 8.0, 300 mM NaCl. Protein solution was mixed at 1:1 ratio with the reservoir solution containing 85 mM Tris-HCl (pH 8.5), 142 mM MgCl_2_, 2.78 M 1,6-hexanediol, 450 mM non-detergent sulfobetaines 195 (NDSB-195). β-galactosidase was crystallized by sitting drop methods, and crystals were grown at room temperature for two weeks. Hexagonal crystals were cryo-protected in buffer containing 0.1 M Tris-HCl (pH 8.5), 167 mM MgCl_2_, 3.27 M 1,6-Hexanediol, 25% glycerol, and flash frozen in liquid nitrogen.

### 2.10. Data Collection, Structure Solution and Refinement

X-ray diffraction data for β-galactosidase were collected at 100 K at the beamlines Proxima 1 at the SOLEIL Synchrotron (France), using a Pilatus 6 M detector. The data were integrated and scaled in XDSme51, and screened against contaminants using the ContaMiner server [39,40]. The crystal structure of β-galactosidase was determined by automated molecular replacement using BALBES [41]. The crystal structure was manually adjusted using Coot and refined using Phenix Refine [42,43].

### 2.11. Molecular Modeling

To generate the missing residues of hla_bga, a multi-chain modelling using SWISS-MODEL was employed [44]. The crystal structure of hla_bga (with missing residues) was used as template structure. The overall structure and stereo chemical quality of the modelled hla_bga was assessed using the SAVES server and QMEANDisco tool from the QMEAN server [45]. The model was evaluated by QMEAN scores, and the QMEANDisco method was used to validate the local quality of the model (Figure S6). The overall global score of the homology model is 0.73 ± 0.05, global score ranges between 0 and 1.

### 2.12. Structural Analysis

We used the available structures from the Protein Data Bank (PDB) database for the selected proteins. Visualization, electrostatic surface potential calculations and solvent accessible area were performed using Adaptive Poisson-Boltzmann Solver (APBS) electrostatics plugin [46] with default parameters [47] from PyMOL 2.1. The units of the electrostatic surface potential in units are kcal (mol·electron)^−1^ (vs. salt-free buffer). Residues whose solvent accessibility (surface exposed amino acids) was greater than 10% were determined using the Swiss PDB viewer 4.1 [48]. The number of hydrogen bonds and salt bridges were calculated with default parameters using Discovery Studio Visualizer (DSV) [49] and Visual Molecular Dynamics (VMD), respectively [50].

### 2.13. Molecular Dynamics Simulations

We prepared the initial model for the molecular dynamics simulations from the hla_bga X-ray structure with modeled missing residues reported in this work. The structures for the hla_bga homologs were taken from the Protein Data Bank (PDB) [51]. As a mesophilic homolog, we used the *Bacillus circulans* sp. *Alkalophilus* structure (PDB ID: 3TTS, resolution 2.4 Å) [52], while, as a thermophilic homolog, we used the *Thermus thermophilus* sp. A4 structure (PDB ID: 1 KWG, resolution 1.6 Å) [53].

We used GROMACS 2018.1 [54,55] for preprocessing the structures, running the molecular dynamics simulation, and analyzing the resulting trajectories. The methodology used for protein modeling, system setup, equilibration, and simulations was similar to that used in previous studies [56]. Simulations were performed using the optimized potentials for liquid simulations (OPLS) force field [57] and the Simple Point Charge (SPC) water model for an explicit water environment [58]. Protein charges were neutralized by adding Na^+^ ions, as required. Proteins were immersed in a box of water molecules large enough to have at least a distance of 10 Å between the protein and the box boundaries. Starting structures were energy minimized using the steepest descent algorithm (5000 steps) while freezing the heavy atoms of the protein. After minimization, each protein was equilibrated by a 500 ps simulation in the Canonical (NVT, moles (N), volume (V) and temperature (T) are conserved) ensemble at 10 °C, followed by a 500 ps simulation in the Isothermal-Isobaric (NPT, moles (N), pressure (P) and temperature (T) are conserved) ensemble at 10 °C. The Nose–Hoover thermostat [59,60] was used to control temperature, while the Parrinello–Rahman barostat [61] was used to control pressure. A particle-mesh Ewald method [62] was used for long-range electrostatic interactions. The LINear Constraint Solver (LINCS) algorithm [63] was used to constrain all bonds involving hydrogen atoms. Dynamics were performed using a 1 fs time step. The equilibrated systems were used as inputs for starting the production runs. In all cases we performed 400 ns long simulations, starting from 10 °C, and increasing the temperature to 27 °C, 47 °C and 72 °C at the frames corresponding to 100, 200 and 300 ns. For each system, the three replicas of the simulations were performed by changing the velocity seed at the beginning of the molecular dynamics equilibration. All the reported analyses were performed on the last 20 ns of each temperature window.

### 2.14. Accession Numbers

Atomic coordinates and structure factors have been deposited in the Research Collaboratory for Structural Bioinformatics (RCSB) Protein Data Bank under ID code 6LVW.

## 3. Results

### 3.1. Recombinant Production and Biochemical Assessment of hla_bga

Expression and purification of haloarchaeal proteins with high yield is difficult because of the necessity of keeping high salinity throughout the process. We solved this problem by cloning the hla_bga gene as an N-terminal hexahistidine fusion protein for expression in the *Haloferax volcanii* expression systems [15,35], a genetically tractable haloarchaeal strain lacking β-galactosidase activity (Table S1, Figures S1 and S2, see Methods). We successfully purified the 700-amino acid protein using a combination of Ni-sepharose affinity chromatography and ultrafiltration under high salt conditions (Methods) and confirmed its identity using mass spectroscopy and matrix-assisted laser desorption ionization time-of-flight mass spectrometry (MALDI-TOF MS) (Methods, Figures S3 and S4).

**Figure 1:**
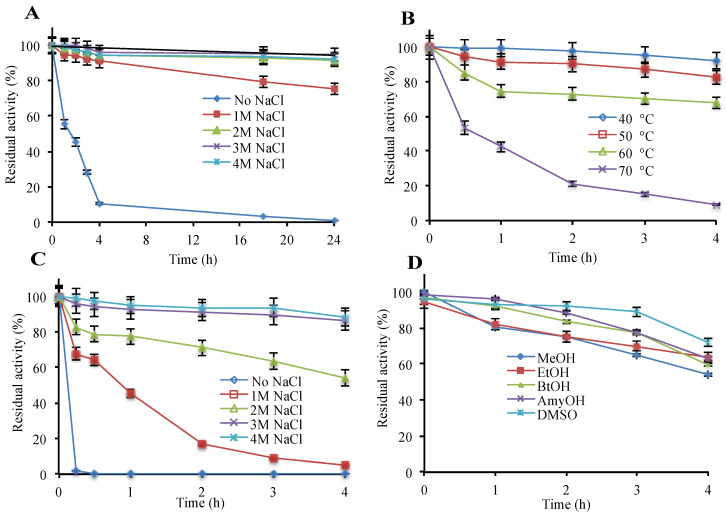
Effect of various parameters on the β-galactosidase stability. (**A**) NaCl (room temperature), (**B**) temperature (4 M NaCl), (**C**) protective effect of salt against temperature (50 °C), (**D**) organic solvents, 20%, v/v and 2 M NaCl (room temperature). Details are given in “Materials and Methods”. As a control, β-galactosidase without additive was used. (100% = 38,209 μmol min^−1^ mg^−1^).

The recombinant enzyme was found to be polyextremophilic, functioning in 0.5 to 4.5 M salt, temperatures from 4 °C to 70 °C, and in the pH range of 6.0–8.0. Highest β-galactosidase activity was observed at 4.0 M NaCl or KCl, 50 °C and a pH of 6.5 (Table S2). The enzyme showed appreciable stability up to 4 M NaCl/KCl and high temperature (60 °C) where the melting temperature (Tm) was 62 °C (Figure 1A,B and Figure S5). Reducing the salt concentration lowered the melting temperature (Tm), and the complete removal of salt entailed irreversible enzyme inactivation within an hour, revealing a strong correlation between salt concentration and enzyme stability (Figure 1A,C and Figure S5). Therefore, for long term stability, the enzyme requires at least 1 M NaCl or KCl. Additionally, hla_bga was stable in the organic solvents, methanol, ethanol, butanol, pentanol, and DMSO up to 20% (v/v) concentration (Figure 1D).

### 3.2. Overall Structure of hla_bga and Subunit Interactions

To understand the molecular features that enable hla_bga to adapt to high salinity/low temperature polyextremophilic conditions, we determined the crystal structure of hla_bga at a resolution of 2.5 Å (Table 1), using the molecular replacement method.

**Figure 2:**
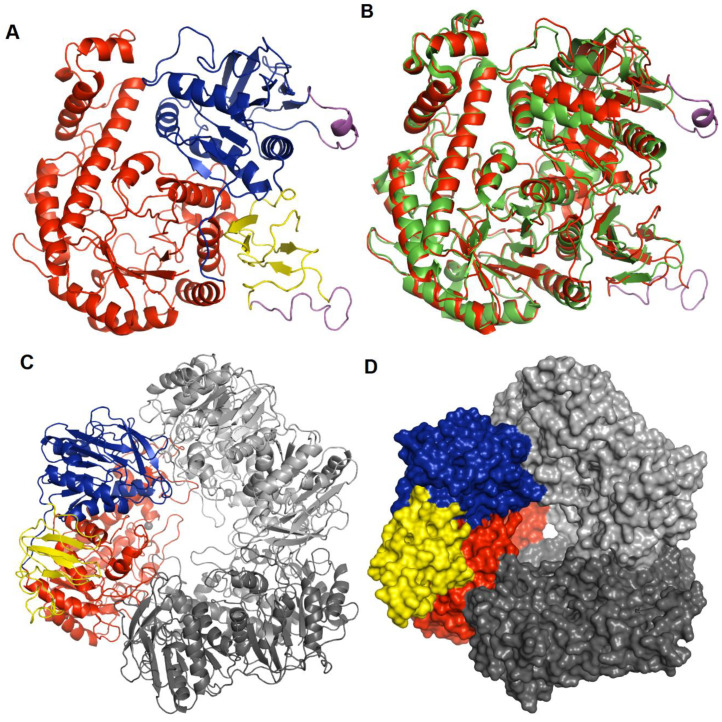
Structure of hla_bga. (**A**) Overall structure of the hla_bga monomer shown as a ribbon model (domains A, red; B, blue; C, yellow). The modelled loop regions not visible in the electron density are shown in magenta color. (**B**) Superimposition of hla_bga (red) and tth_bga (green). (**C**) Trimeric model structure of hla_bga, the domains of one monomer is shown as in Figure 2A while the other two domains are shown in light and dark grey (**D**) The surface area of trimer structure of hla_bga, one monomer is colored as in Figure 2A while the other two domains are shown in light and dark grey.

Hla_bga forms a homo-trimer where each monomer contains three domains: domain A, an (β/α)8 TIM barrel fold domain (residues 1–390); domain B, an α/β fold structure with five helices and eleven β-strands (391–614); and domain C, an anti-parallel β-sandwich structure comprising four β-strands and one helix (615–700) (Figure 2A). Electron density was missing for 32 residues in three regions (residues 532–543, 657–672, and 697–700). These missing residues were modelled in cases where a full-length model was needed for our analysis (Figure 2, Figure S6). As a trimer, hla_bga resembles a bowl with a central opening that has a width of about 25 Å at the rim, ~40 Å in the center and 7 Å at the bottom. Based on hydrophobic cluster analysis, amino acid sequence similarities, the conservation of catalytic residues and reaction mechanisms, hla_bga is classified as a member of the glycoside hydrolase (GH) family 42 [64] (Figure S7).

### 3.3. Catalytic Site Architecture

The structurally and functionally characterized homolog with the highest sequence identity to hla_bga is the β-galactosidase from an extreme thermophile, *Thermus thermophilus* A4 [53] (tth_bga; PDB ID: 1KWG, Figure 2B, Tables S3 and S4). The T. thermophilus domain A, which contains the β-d-galactose-binding site, is also the closest structural match to hla_bga domain A (according to the Distance-matrix ALIgnment (DALI) server [65]; Figure 2B, Table S4). Tth_bga has 11 direct hydrogen bond interactions between the protein residues (1KWG numbering: Arg102, Asn140, Glu141, Tyr266, Glu312, Trp320, Glu360, and His363) and the OH groups of galactose, as well as a hydrophobic interaction with Phe350 and several other water-mediated hydrogen bonds [53]. The sequence and structure comparison of the galactose-bound tth_bga (1KWG) with hla_bga showed that all the catalytic residues were conserved and perfectly overlapped near the binding site of the ligand (Figures S7 and S8), indicating that the galactose binding modes of both enzymes are the same.

### 3.4. Quaternary Structure

The three monomers of hla_bga are tightly bound to each other by extensive hydrogen bonding. Each monomer has two contact areas with its nearby chains—one from the TIM barrel of domain A and the other from α/β fold of domain B, whereas domain C is located at the rim of the bowl. Each chain of hla_bga has 18 hydrogen bonds and four salt bridges that contribute to the inter-chain interactions (Figure 2C) The solvent-accessible surface area buried in the trimer structure of hla_bga is 10,083 Å2, which constitutes 43.1% of the trimer surface area (Figure 2D). Although proteins from psychrophiles are usually denatured at elevated temperatures, hla_bga showed relatively good stability even at 60 °C (Figure 1B). These extensive inter-chain hydrogen bond interactions may contribute to the stability of hla_bga.

### 3.5. Hla_bga Combines Adaptive Features for Halo- and Psychrophily

To understand the mechanism for the dual salt and cold adaptation, we compared hla_bga with those homologous GH family 42 bga enzymes for which the structure was available (four from mesophiles, *Bacillus circulans* sp. *Alkalophilus*, *Bifidobacterium animalis* subsp. lactis Bl-04, *Bifidobacterium* species, *Bifidobacterium bifidum* S17; two from thermophiles, *Thermus thermophilus* A4, *Geobacillus stearothermophilus*; and two from psychrophile, *Rahnella* sp. R3, *Marinomonas* ef1). The structure of ß-galactosidase from *Bifidobacterium* adolescentis (PDB ID: 5VYM), although belonging to family 42, was excluded because it is only a 232-residue fragment. Although crystal structures of halophilic β-galactosidases of family 42 were not available, we included the available β-galactosidase sequences of family 42 from halophilic archaea in our analysis (Table 2). Additionally, to find a general trend of halophilic, thermophilic, mesophilic, and psychrophilic proteins, we compared the hla_bga sequence and structure with non-β-galactosidase proteins (Table S5).

**Figure 3:**
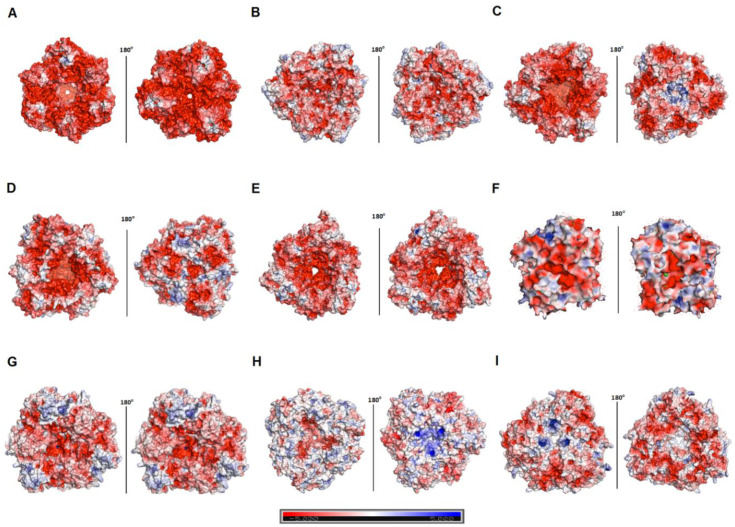
Surface charge of various β-galactosidases. (**A**–**I**) Surface charge of the β-galactosidase from *H. lacusprofundi* (hla_bga) model and its structural homologs. Red and blue colors represent negative and positive charges of the protein. (**A**) *Halorubrum lacusprofundi*, hla_bga (**B**) *Bacillus circulans* sp. *Alkalophilus*, (PDB ID: 3TTS) (**C**) *Bifidobacterium animalis*, (PDB ID: 4UNI) (**D**) *Bifidobacterium bifidum* S17, (PDB ID: 4UZS) (**E**) *Bifidobacterium* species, (PDB ID: 5XB7) (**F**) *Marinomonas* ef1, (PDB ID: 6Y2K) (**G**) *Rahnella* sp. R3, (PDB ID: 5E9A) (**H**) *Thermus thermophilus* A4, (PDB ID: 1KWG) (**I**) *Geobacillus stearothermophilus*, (PDB ID: 4OIF). Units: kcal (mol.electron)^−1^ (vs. salt free buffer).

Sequence comparison between hla_bga and other family 42 members revealed a significant level of diversity even among mesophiles and thermophiles (Table 2, Figures S7 and S9). However, hla_bga displayed clear features associated with halophily. Firstly, hla_bga is highly acidic, having a 5–7% excess of acidic residues over basic residues compared to psychro-, meso- and thermophilic homologs (Table 2). This charge imbalance helps protein solvation at high salinity [14]. In hla_bga it is caused by the lower number of lysines and a higher number of aspartic and glutamic acids compared to its homologs. It is particularly pronounced at the hla_bga surface, displaying 33.6% acidic residues (Table 2 and Figure S11). In contrast, thermophilic bga and psychrophilic bga have only 24.3% and 17.2% of acidic surface residues, respectively, whereas mesophilic homologues average 25.6%. Interestingly, in the case of bga from *Marinomonas* ef1, the number of acidic residues was comparatively low (23%) even though it was also sourced from Antarctica [66]. Concomitantly, there are less positively charged surface residues on hla_bga than observed for psychrophilic, mesophilic and thermophilic homologs (14.8% vs. 17.3%, 20.5% and 23.7%, respectively) (Table 2, Figure 3 and Figure S11).

Secondly, hla_bga also showed a reduction in protein hydrophobicity, a halophilic feature to counterbalance the increased hydrophobic effect at high salinity. Compared to its homologs, hla_bga contains a higher content of borderline hydrophilic–hydrophobic residues such as serine and threonine that reduce the overall hydrophobicity [67] of the protein (−0.55 vs. −0.34, −0.35, −0.36 and −0.50 in case of psychro, meso- and thermophilic and halophilic β-galactosidases, respectively, Table 2, Figure S9). Additionally, on the hla_bga protein surface, lysine residues (1.0%) are massively replaced by arginine residues (11.7%), in stark contrast to the protein surface ratio of lysine vs. arginine in homologs from thermophiles (7.7% vs. 13.5%), mesophiles (5.7% vs. 11.2%) and psychrophiles (5.8% vs. 7.5%). The reduction in lysine residues helps halophilic proteins to reduce their hydrophobic surface (contributed by the associated alkyl component of the lysine side chain) in addition to increasing the overall negative charge on the surface [25,68].

Besides the signature electrostatic profile of salt adaptation, hla_bga also showed features distinct from their homologs which may enhance the structural flexibility of the protein to enable the conformational dynamics required for catalysis at extremely low temperatures. Compared to its homologs, hla_bga has a higher number of small amino acids (glycine and alanine, 18.9% vs. 16.7%, 17%, 16.2% and 18.6% for psychro-, meso-, thermo- and halophilic β-galactosidases, respectively). Moreover, a smaller fraction of residues forming α-helices and β-strands were found in hla_bga compared to its homologs. Moreover, the total number of inter and intra-hydrogen bonds in hla_bga were lower compared to its mesophilic, psychrophilic, and thermophilic homologs, compensating for the marginally higher number of intra-monomer salt bridges (Table 2). Finally, hla_bga contains two unusual surface loop regions, one in domain B (residues 532–543) and the other in domain C (656–674). Electron density for these loops was missing, inferring that they were highly flexible. These two loop regions contained an increased number of acidic amino acids (42% in the loop region compared to 18.6% overall protein structure), illustrating the convolution of halophilic and psychrophilic features. Interestingly, hla_bga has an increased number of prolines, even compared to thermophilic β-galactosidases (Table 2). Prolines rigidify the structure of proteins and enhance their thermostability for entropic reasons, which correlates well with the higher thermal stability of the hla_bga enzyme even though the enzyme’s source organism is found in psychrophilic conditions [69,70].

### 3.6. Molecular Dynamics Simulations Show Residue-Level Flexibility

Our analysis suggested that hla_bga has evolved a higher flexibility as a means of maintaining catalytic turnover at low temperatures. To investigate the flexibility of hla_bga compared to homologous proteins, we used molecular dynamics (MD). We performed three independent 100-ns MD simulations at four temperatures, 10 °C, 27 °C, 47 °C and 72 °C, for the monomer of hla_bga, of its thermophilic homolog tth_bga, and of its mesophilic homolog from *Bacillus circulans* (bci_bga; PDB ID: 3TTS). We also performed control simulations on the hla_bga trimer. All the simulations converged, as shown by the corresponding root mean square deviation (RMSD) plots (Figure S12).

**Figure 4:**
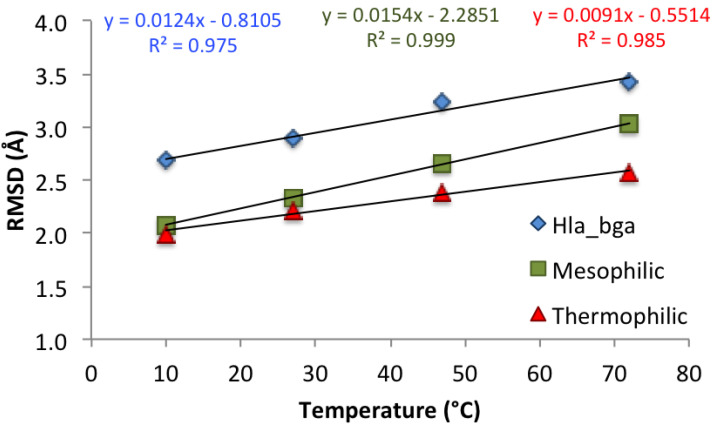
Effect of temperature on the protein backbone root mean square deviation (RMSD) for the three simulated systems. Values are averaged over the last 20 ns of the three independent simulations for each system. Corresponding trend lines are shown with relative correlation coefficients (R2) and equations.

As shown recently [71], the best descriptor of differences in global protein stability among homologs is the change in backbone RMSD with rising temperature, rather than its absolute value, because RMSD changes reflect the flexibility acquired by the system as a consequence of the temperature increase. RMSD values (each presenting an average of the last 20-ns of the three simulations) at increasing temperatures for hla_bga and its homologs showed correlation coefficients (R2) of around 0.98 (Figure 4). As expected, the flexibility of the protein monomers increased with temperature for all three systems as quantified by the RMSD. Control simulations run for the hla_bga trimer showed the same trend as observed for the hla_bga monomer (Figure S13), thus validating the use of the monomer simulations to infer protein dynamics. As expected, based on the negative correlation between temperature adaptation and heat-induced increase in backbone flexibility demonstrated by Dong et al. [71], the thermophilic tth_bga was least sensitive to the temperature increase as illustrated by the overall lowest RMSD changes (with a regression line slope of 0.0091). Hla_bga was more sensitive than its thermophilic homolog (regression line slope of 0.0124) but less sensitive than the mesophilic bci_bga (regression line slope of 0.0154) to the rising temperature. This last result would be unexpected based on the psychrophilic nature of hla_bga only, but matched our experimental finding that hla_bga was thermostable with a Tm of 62 °C.

**Figure 5:**
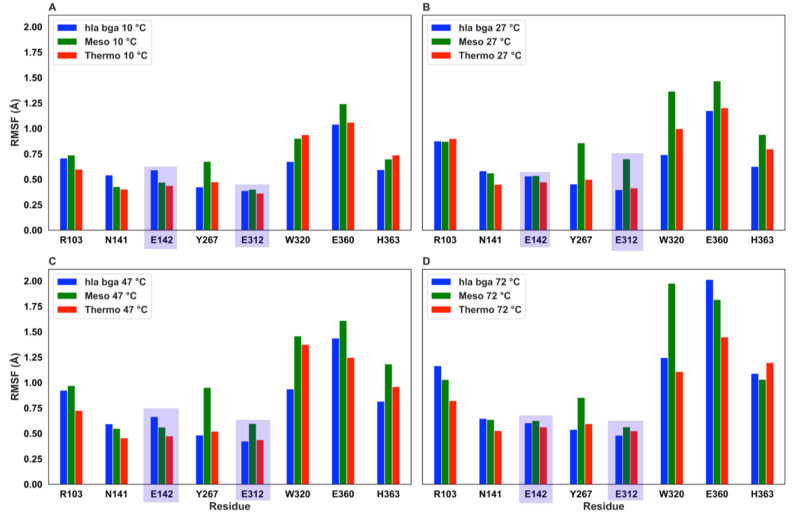
Root mean square fluctuation (RMSF) values at increasing temperatures for the two catalytic residues (evidenced by a purple shadow) and the six conserved substrate-binding residues. The RMSF was obtainted from MD simulation at (**A**) 10 °C, (**B**) 27 °C, (**C**) 47 °C and, (**D**) 72 °C.

To investigate the effect of increasing temperature on the residue-specific flexibility, we also calculated the root mean square fluctuation (RMSF) values (Figure S14). The RMSF exhibited similar values and trends for the three homologs, with the hla_bga presenting, however, distinctive peaks in residues of the 532–543 and 657–672 loops, in agreement with our crystallographic observations. To further investigate the heat effect locally for functionally important residues, we analyzed the RMSF values for the eight residues being either catalytic (Glu142, Glu312, hla_bga numbering) or forming the substrate-binding pocket (Arg102, Asn140, Tyr266, Trp320, Glu360 and His363; Figure 5).

As expected, all values generally increase with temperature. The fluctuation of the two catalytic residues (Glu142 and Glu312) was reduced for all proteins under all temperatures investigated, indicating that the molecules had evolved dynamic features that allowed the preservation of the spatial position of the catalytic residues to maintain catalysis. Overall all eight residues fluctuated markedly more in the mesophilic homolog than in the thermophilic homolog and in hla_bga. Moreover, considering the changes in the residue RMSF at rising temperature, we observed that for temperatures increasing from 10 °C to 47 °C none of the eight residues (catalytic or substrate-binding pocket residues) in hla_bga and in the thermophilic homolog reach the significance threshold of 0.5 Å (proposed by Dong [71] et al. for a similar temperature rise), while one residue in the mesophilic homolog does (Trp320, being Trp315 in the bci_bga mesophile numbering, see Table S6). Therefore, also locally, hla_bga exhibited an intermediate flexibility, as compared to the other two investigated systems. Its catalytic and binding residues are held in place more firmly at high temperatures than in the mesophilic homolog, a finding that correlates with the catalytic activity at elevated temperatures we observed experimentally for hla_bga.

## 4. Discussion

Our analysis of *H. lacusprofundi* β-galactosidase revealed a polyextremophilic enzyme with activity and stability under extreme temperatures and at high concentrations of salt and organic solvents. Hence, hla_bga represents a valuable model for analyzing enzyme adaptation to extreme environments, especially because to the best of our knowledge only two polyextremophilic enzyme structures have been reported [64,72,73], and no crystal structure of a salt-adapted β-galactosidase of family 42 has yet been determined.

Hla_bga displayed hallmarks of both salt and cold adaptation. Halophily was evidenced by a highly negatively charged surface [14,20,29]. The negatively charged surface residues coordinate hydrated ions and therefore form a highly ordered solvation shell, which prevents aggregation and increases protein solubility [15,25,74,75]. Additionally, halophilic proteins have a reduced overall hydrophobicity to prevent aggregation and retain structural flexibility at high salt concentrations [76]. Indeed hla_bga displayed signs of surface and internal reduction of hydrophobic moieties.

To function in cold conditions, proteins contain features to enhance their conformational dynamics for low-temperature catalysis. As a distinct feature for psychrophily, hla_bga contains two unusually long and flexible loops (Domain B, 532–543; Domain C, 656–674) (Figure 4 and Figure S10). Flexible loops increase protein dynamics and allow a protein to adopt multiple conformations at low temperatures [77,78,79,80,81,82], whereas loop trimming is frequently used to enhance the structural rigidity of a protein [81]. Additionally, hla_bga also harbored less stabilizing secondary structures, fewer intra- and intermolecular hydrogen bonds and more small and flexible residues than mesophilic orthologs.

However, hla_gba also revealed atypical additional features which may reflect compromises to achieve polyextremophily. For example, to achieve surface acidity (salt adaptation), hla_bga displays more aspartic acids (21.5%) than glutamic acids (12.1%) on its surface. However, halophilic proteins prefer glutamic acids rather than aspartic acids on their surface because glutamic acids bind more water at physiologic pH than aspartic acids do [25,83]. Furthermore, hla_bga has an even higher number of prolines than thermophiles, presumably because they break secondary structures (cold adaption) and can replace large hydrophobic residues inside the core (salt adaptation). However, prolines also rigidify the structure of proteins which is unwanted in cold environments, but used in thermophiles (Table 2). Similarly, hla_bga has a relatively large number of intra- and intermolecular salt bridges. A higher number of salt-bridges was observed in the halophilic malate dehydrogenase from Haloarcula marismortui and glucose dehydrogenase from Haloferax mediterranei which enhanced the enzyme stability at high salt concentrations compared to its mesophilic homologs [25,83,84]. Salt bridges also stabilize proteins as an adaptation to high temperature [83,85], whereas psychrophilic proteins tend to have fewer salt bridges and achieve higher flexibility at the cost of reduced stability. Thus, the biologically unnecessary heat stability that we observed for hla_bga appears to be the side-effect of dual cold and salinity adaptation.

Collectively, our study of hla_bga from Deep Lake of Antarctica illustrates how this protein combines features for both, halophily and psychrophily within the same structure. This combination is achieved through a convolution of features, when they are compatible, such as amino acid composition and with a high number of acidic residues. If the features are incompatible, they are compensated, by for example higher amounts of salt bridges with fewer hydrogen bonds. These compensatory mechanisms might have given rise to the additional unexpected thermal stability. Our study may provide a basis for designing polyextremophilic enzymes with industrially valuable properties, and help reveal mechanisms of protein evolution and adaptation under extreme and potentially astrobiological conditions.

## Figures and Tables

**Table 1:** Data collection and refinement statistics.

Resolution Range	32.78–2.49 (2.58–2.49)
Space group	P 63
Unit cell	100.13 100.13 137.43 90 90 120
Total reflections, unique reflections	539,035 (43,095), 27,088 (1673)
Multiplicity	19.9 (16.5)
Completeness (%)	86.53 (61.47)
Mean I/sigma (I)	15.40 (0.64)
Wilson B-factor	75.69
R-merge, R-meas, R-pim	0.1509 (4.187), 0.1549 (4.319), 0.03468 (1.045)
Correlation coefficient, CC1/2, CC	0.999 (0.556), 1 (0.845)
Reflections used in refinement and for R-free	23,592 (1672), 1230 (103)
Reflections used	
R-work, R-free	0.2457 (0.4856), 0.3080 (0.5417)
CC (work), CC (free)	0.895 (0.077), 0.855 (0.192)
Number of non-hydrogen atoms	5280
Macromolecules, ligands, solvent	5275, 2, 3
Protein residues	668
Root-mean square (bonds, angles)	0.005, 0.98
Ramachandran favored, allowed, outliers (%)	92.75, 7.1, 0.15
Rotamer outliers (%)	0
Clash score	23.11
Average B-factor	100.99
Macromolecules, ligands, solvent	101.01, 93.91, 66.24
Number of translation/libration/screw (TLS) groups	1

Statistics for the highest-resolution shell are shown in parentheses.

**Table 2:** Sequence and structural analysis of β-galactosidases.

**Sequence analysis**		**hla_bga**	**Psychro**	**Meso**	**Thermo**	**Halo**
	Isoelectric point (pI)	4.4	5.7	5.1	5.8	4.5
	Grand average hydrophobicity	−0.55	−0.34	−0.35	0.36	−0.5
	Aliphatic amino acids, Ala, Ile, Leu, Val (%)	27.5	28.6	28.4	29.4	27.8
	Positively charged amino acids, R, K and H (%)	11.4	13.1	12.2	14.3	11.2
	Negatively charged amino acids, D and E (%)	18.6	12.2	13.9	13.5	17.5
	Small amino acids, G and A (%)	18.9	16.7	17.0	16.2	18.6
	Hydrophobic residues, F, I, L, V, M (%)	21.4	25.9	25.5	27.0	22.0
	Polar uncharged residues (%)	14	18.2	18.2	14.2	15.7
	Non-polar residues (%)	45.6	46.1	44.3	45.4	44.8
	Aspartic acids (%)	11.7	5.8	7.6	5.2	10.1
	Glutamic acids (%)	6.9	6.5	6.3	8.3	7.4
	Aromatic residues (%)	10.4	10.9	11.3	12.7	10.8
	Proline amino acids (%)	7.1	5.4	4.6	6.3	6.2
	Arginine amino acids (%)	7.7	5.7	5.9	7.4	7.4
**Structural analysis**	Positive residues among total number of surface residues (%)	14.8	17.3	20.5	23.7	NA
	Negative residues among total number of surface residues (%)	33.6	17.2	25.6	24.3	NA
	Polar residues among total number of surface residues (%)	12.7	20.2	18.8	13.0	NA
	Non-polar residues among total number of surface residues (%)	34.9	38.2	29.8	33.2	NA
	Aromatic residues among total number of surface residues (%)	3.9	7.4	5.4	5.9	NA
	Amino acids that form helices (%)	30.4	36.9	32.3	33.5	NA
	Amino acids that form strands (%)	17.7	19.9	22.3	21.0	NA
	Amino acids with bulky hydrophobic side chains (F, I, L) in the protein surface (%)	5.5	11.8	5.0	9.4	NA
	Hydrogen bonds (all H-bonds and inter-chain H-bonds)	663, 18	771, 23	805, 25	778, 23	NA
	Salt bridges (all salt bridges and inter-chain salt bridges)	44, 4	40, 3	43, 4	50, 6	NA

The above values represent the average score of β-galactosidases. For sequence analysis, the amino acid sequence of hla_bga was compared with available β-galactosidase sequences of family 42 from halophilic archaea: (http://www.cazy.org/GH42_archaea.html). NA—not available (there are no structures available for halophilic β-galactosidase sequences of family 42).

## Supplementary Materials

The following are available online at https://www.mdpi.com/2076-2607/8/10/1594/s1, Figures S1–S14; Tables S1–S6.
