# Supplementary material for: Understanding High-Salt and Cold Adaptation of a Polyextremophilic Enzyme
Source: Microorganisms. 2020 Oct 16;8(10):1594. doi: 10.3390/microorganisms8101594 (PMC7602713; doi:10.3390/microorganisms8101594)
Supplement: Supplementary file 1 [file microorganisms-08-01594-s001.pdf]

Supplementary information

Understanding high-salt and cold adaptation of a polyextremophilic enzyme

Ram Karan[1*], Sam Mathew[1], Reyhan Muhammad[2], Didier B. Bautista[1], Malvina Vogler[1], Romina Oliva[1,3], Luigi Cavallo[1], Stefan T. Arold[2,4*], Magnus Rueping[1*]

[1]King Abdullah University of Science and Technology (KAUST), KAUST Catalysis Center, Thuwal 23955-6900, Saudi Arabia

[2]King Abdullah University of Science and Technology (KAUST), Computational Bioscience Research Center (CBRC), Biological and Environmental Science and Engineering (BESE), Thuwal, 23955-6900, Saudi Arabia

[3]Department of Sciences and Technologies, University Parthenope of Naples, Centro Direzionale Isola C4, I-80143 Naples, Italy

[4]Centre de Biochimie Structurale, CNRS, INSERM, Université de Montpellier, 34090 Montpellier, France

[*]Corresponding author: Ram Karan, ram.karan@kaust.edu.sa, Stefan T. Arold, stefan.arold@kaust.edu.sa; Magnus Rueping, magnus.rueping@kaust.edu.sa

Index:

Table S1. Oligonucleotides used in this study

Table S2: Biochemical properties of crystalized β-galactosidases from GH42 family.

Table S3. β-galactosidase sequence identity matrix (Family 42)

Table S4. Structural homologs (DALI) of β-galactosidase from *Halorubrum lacusprofundi*

Table S5. Trends for halo-, psychro and thermoadaption compared mesophilic proteins

Table S6. Differences in residue RMSF values (ΔRMSF) between 47 and 10 °C for eight functionally relevant residues.

Figure S1. Shuttle vector for the expression of bga in *Haloferax volcanii*

Figure S2. X-gal hydrolysis

Figure S3. Coomassie and InVision His-tag In-gel stained SDS-PAGE, tryptic digest and LC-MS/MS analysis of β-galactosidase

Figure S4. MALDI-TOF analysis of purified β-galactosidase

Figure S5. Measured melting curve and fitting curve

Figure S6. Structural assessment of hla_bga homology model produced by Swiss Modeller

Figure S7. Sequence alignment based on Hidden Markov Model (HMM) profiles, obtained with HHPRED
Figure S8. β-D-galactose binding pocket.

Figure S9. Ratio of amino acids in hla_bga and its structural homologs

Figure S10. Superimposed β-Galactosidase monomers.

Figure S11. Ratio of amino acids on the solvent-exposed surface in the hla_bga and its structural homologs.

Figure S12. Backbone RMSD plots over time for the three independent simulations of the three investigated systems at the four explored temperatures (10, 27, 47 and 72 °C).

Figure S13. Effect of temperature on the hla_bga monomer and trimer backbone RMSD.

Figure S14. RMSF values averaged over the last 20 ns of the three simulations ran for each investigated system at 10, 27, 47 and 72 °C.

Table S1. Oligonucleotides used in this study

**Primer**	**5′-3′sequence**	**Use**
7His.bga F	cggcccaacggttgattgcatatgcaccaccaccaccacca ccacATGCGTCTCGGAGTCTGTTAC	Inserting *bga* gene and 6His
7His.bga R	ggtcgactctagaactagtggatcCTACGTCTGAG CGATTCTC<br>CCACCACCATATGATGATG	
pTA693.F	GTTCGAACCGCCCTTTCC	Sequencing of *bga* gene
pTA693.R	ATGACCATGATTACGCCAAG	

Table 2: Biochemical properties of crystalized β-galactosidases from GH42 family.

PDB ID	Organism	Type	Temperature range, optima (°C)	pH range, optima	Salt range, optima (M)
[1]hla_bga (current work)	*Halorubrum lacusprofundi*	Polyextremophiles	4-70, 50	6.0-8.0, 6.5	0.5-4.5, 4
[2,3]1KWG	*Thermus thermophilus* A4	Thermophile	40-90, 70	5.0-9.0, 6.5	-
[4,5]5E9A	*Rahnella* sp. R3	Psychrophile	4-45, 35	5.5-8.0, 6.5	-
[6]4UZS	*Bifidobacterium bifidum* S17	Mesophile	20-55, 40	4.0-8.0, 6.0	-
[7]3TTS	*Bacillus circulans* sp. *alkalophilus*	Mesophile	10-70, 55	4.5-10, 6.5	-
[8,9]4OIF	*Geobacillus stearothermophilus*	Thermophile	30-80, 60	4.5-10, 6.0	-
[10]4UNI	*Bifidobacterium animalis* subsp. *lactis* Bl-04	Mesophile	30–70, 37	4.0-10, 6.5	-
[11]5XB7	*Bifidobacterium* species	Mesophile	37	6.5	-
[12]6Y2K	*Marinomonas* ef1	Psychrophile	55	6.0	0-2.4

Salt range optima of β-galactosidases other than hla_bga and 6Y2K are not available.

Table S3. β-galactosidase sequence identity matrix using Clustal Omega (Family 42)

	**hla_bga**	**1KWG**	**6Y2K**	**4UNI**	**4UZS**	**3TTS**	**4OIF**	**5E9A**	**5XB7**	**5VYM**
**hla_bga**	100	46	41	29	29	29	29	28	26	24
**1KWG**	46	100	55	29	31	31	29	33	26	21
**6Y2K**	41	55	100	27	27	29	27	29	27	20
**4UNI**	29	29	27	100	62	33	30	30	24	30
**4UZS**	29	31	27	62	100	35	32	33	22	37
**3TTS**	29	31	29	33	35	100	40	46	24	27
**4OIF**	29	29	27	30	32	40	100	49	23	25
**5E9A**	28	33	29	30	33	46	49	100	24	28
**5XB7**	26	26	27	24	22	24	23	24	100	23
**5VYM**	24	21	20	30	37	27	25	28	23	100

Halorubrum lacusprofundi, hla_bga; *Bacillus circulans* sp. Alkalophilus, 3TTS; *Bifidobacterium animalis* subsp. lactis Bl-04, 4UNI; *Bifidobacterium bifidum* S17, 4UZS; *Bifidobacterium adolescentis*, 5VYM; *Bifidobacterium* species, 5XB7; *Marinomonas* ef1, 6Y2K; *Rahnella* sp. R3, 5E9A; *Thermus thermophilus* A4, 1KWG; *Geobacillus stearothermophilus*, 4OIF.

Table S4. Structural homologs (DALI) of β-galactosidase from *Halorubrum lacusprofundi*

#	PDB ID	Organism	Type	Z-score	Rmsd (A°)	Identity (%)
1	1KWK-A	*Thermus thermophilus* A4	Thermophile	47.1	1.5	46
2	1KWG-A	*Thermus thermophilus* A4	Thermophile	46.4	1.6	46
3	6Y2K-A	*Marinomonas* EF1	Thermophile	46.4	1.5	41
4	5E9A-A	*Rahnella* sp. R3	Psychrophile	41.3	1.8	28
5	4UCF-A	*Bifidobacterium bifidum* S17	Mesophile	40.6	2.2	29
6	4UZS-A	*Bifidobacterium bifidum* S17	Mesophile	40.3	2.3	29
7	3TTS-D	*Bacillus circulans* sp. *alkalophilus*	Mesophile	40.0	1.9	29
8	3TTY-E	*Bacillus circulans* sp. *alkalophilus*	Mesophile	39.9	1.9	29
9	4OIF-B	*Geobacillus stearothermophilus*	Thermophile	39.1	2.0	29
10	4UOZ-C	*Bifidobacterium animalis subsp. lactis Bl-04*	Mesophile	38.6	2.2	29

Table S5. Trends for halo-, psychro-, and thermoadaption compared to mesophilic proteins

		hla_bga	Halophilic[1]	Psychrophile[2]	Thermophilic[3]
Sequence analysis	pI	decreased	decreased	Decreased	slightly decreased
	Grand average hydrophobicity	increased	increased	Increased	increased
	Aliphatic amino acids (%)	decreased	slightly increased	slightly decreased	increased
	Positively charged amino acids, R, K and H (%)	slightly increased	slightly increased	Decreased	increased
	Negatively charged amino acids, D and E (%)	increased	increased	Decreased	increased
	Small amino acids, G and A (%)	increased	increased	Decreased	decreased
	Hydrophobic residues, F, I, L, V, M (%)	decreased	slightly decreased	Increased	increased
	Polar residues (%)	decreased	decreased	Increased	decreased
	Non-polar residues (%)	decreased	decreased	Decreased	slightly increased
	Aspartic acids (%)	decreased	decreased	Decreased	increased
	Glutamic acids (%)	increased	increased	slightly increased	increased
	Aromatic residues (%)	increased	decreased	slightly increased	increased
	Proline amino acids (%)	increased	decreased	Decreased	decreased
	Arginine amino acids (%)	increased	increased	slightly increased	increased
	Positive residues among total number of surface residues (%)	decreased	decreased	slightly increased	increased
Structural analysis	Negative residues among total number of surface residues (%)	increased	increased	Increased	increased
	Polar residues among total number of surface residues (%)	decreased	decreased	Increased	decreased
	Non-polar residues among total number of surface residues (%)	decreased	decreased	Decreased	decreased
	Aromatic residues among total number of surface residues (%)	decreased	decreased	slightly increased	increased
	Amino acids that form helices (%)	decreased	increased	increased	increased
	Amino acids that form strands (%)	decreased	decreased	decreased	increased
	Amino acids with bulky hydrophobic side chains (F, I, L) in the protein surface (%)	decreased	decreased	increased	increased
	Hydrogen bonds (intra and inter)	increased	decreased	increased	decreased
	Salt bridges	increased	decreased	decreased	decreased

The thermophilic, psychrophilic, halophilic and mesophilic enzymes of varying length were used for the analysis which was reflected in the lower hydrogen bonds and salt bridges compared to hla_bga [13],[14],[15].

Table S6. Differences in residue RMSF values (ΔRMSF) between 47 and 10 °C for eight functionally relevant residues.

**ΔRMSF (Å) between 47 and 10 °C**			
**Residue[a]**	**Hla_bga**	**Mesophile (3TTS)**	**Thermophile (1KWG)**
**R102**	0.216	0.232	0.127
**N140**	0.052	0.121	0.053
**E141**	0.074	0.091	0.037
**Y266**	0.059	0.276	0.047
**E312**	0.036	0.196	0.074
**W320**	0.261	**0.556[b]**	0.436
**E360**	0.396	0.367	0.187
**H363**	0.222	0.484	0.221
**average**	0.164	0.290	0.148

[a] Numbering from hla_bga, [b] Values above the threshold for significance (0.5 Å) indicated by Dong *et al*. 2018 for a similar temperature rise (15 to 42 °C) are in bold.

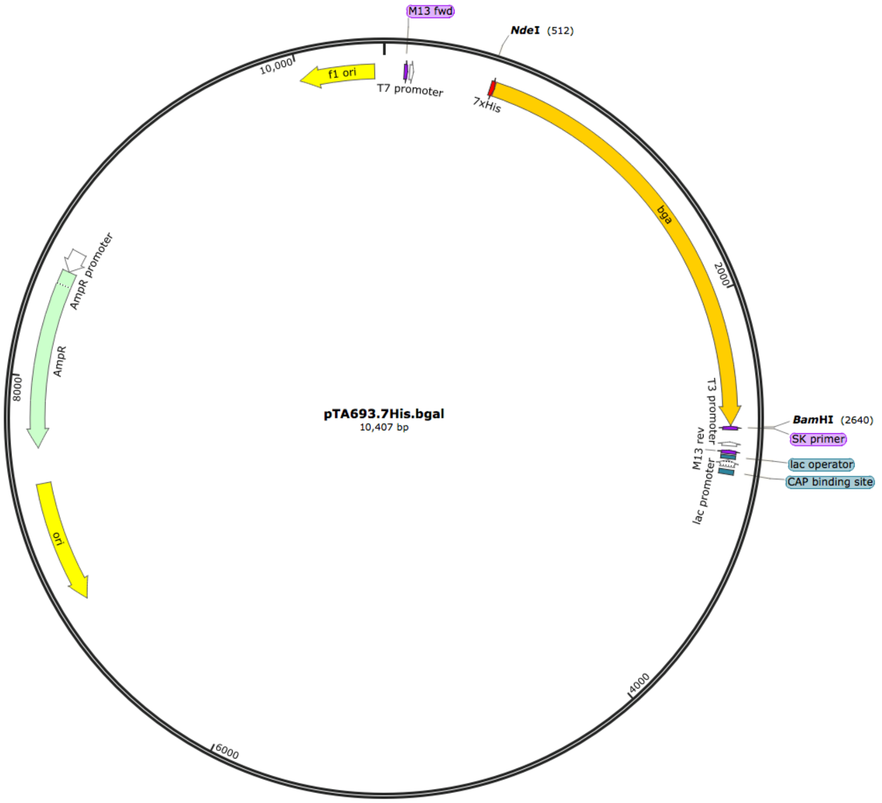

Figure S1. Shuttle vector for the expression of *bga* in *Haloferax volcanii*. The *Nde*I and *Bam*HI restriction sites were used to introduce the *bga* gene.

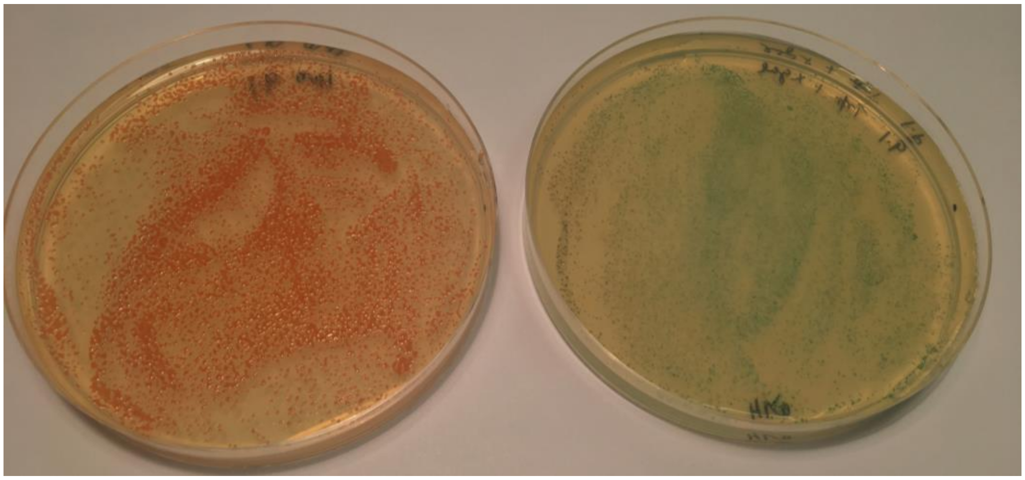

Control β-galactosidase transformants

Figure S2. X-gal hydrolysis. When the β-galactosidase gene is in a gene fusion with the pTA693 vector in *Haloferax volcanii*, X-gal is hydrolyzed indicating the bga protein is produced.

A **B**

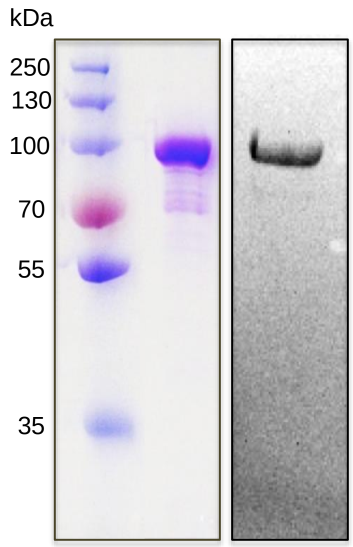

M**RLGVCYFPE HWPSEEWERD VAAMADAGLE**
YVRMAEFSWG VLEPERGTFD FEWLDEAIEL
IGDHGMQAVL CTPTATPPK**W LVDERPSIRQ**
EDPDGTVREH GSRRHYCFNS DAYREETARI
VERVTER**YAD SPHVAGWQTD NEFGCHETVR**
CYCDDCADAF RTWLADRYGD IDRLNEAWGN
AFWSQQYGSF DEIDPPGPTP AEHHPSRLLA
YAR**FSSDSVV EYNRLHADLI READPDWFVT**
HNFMGRFPTL NAYDVSEDLD RVAWDSYPTG
FVQDRYDGEA SPDQLRAGDP DQVGMDHDIY
RSALDRPFWW MEQQPGDVNW PPHCPQPGEG
AMRLWAHHAA AHGADAVLYF RWR**RCLEGQE**
QYHAGLRKAD GSPDRGYADA AHTSEEFATL
DGASHVDAPV AVVFDYDSLW ALNAQPHAPD
FDYWALQEAF YGAVRGR**GVQ VDVVPPSADL**
SGYAAVVAPA LHLVTEDLAD RLTDYIAGGG
EVLFGPRTGV KDAENK**LRPM SQPGPLTDLV**
GATVDQHESL PRRLETTVRR VGDPTDDSEE

Figure S3. (A) Coomassie (left) and **(B)** InVision His-tag In-gel stained (right) SDS-PAGE of purified β-galactosidase. The dominant band of β-galactosidase is visible at ~100 kDa. The protein was purified by IMAC at high salt concentrations (2 M NaCl) including 10 % (v/v) glycerol. (B) Tryptic digest and LC-MS/MS analysis of β-galactosidase. Sequence coverage: 70 %, matched peptides shown in **bold.**

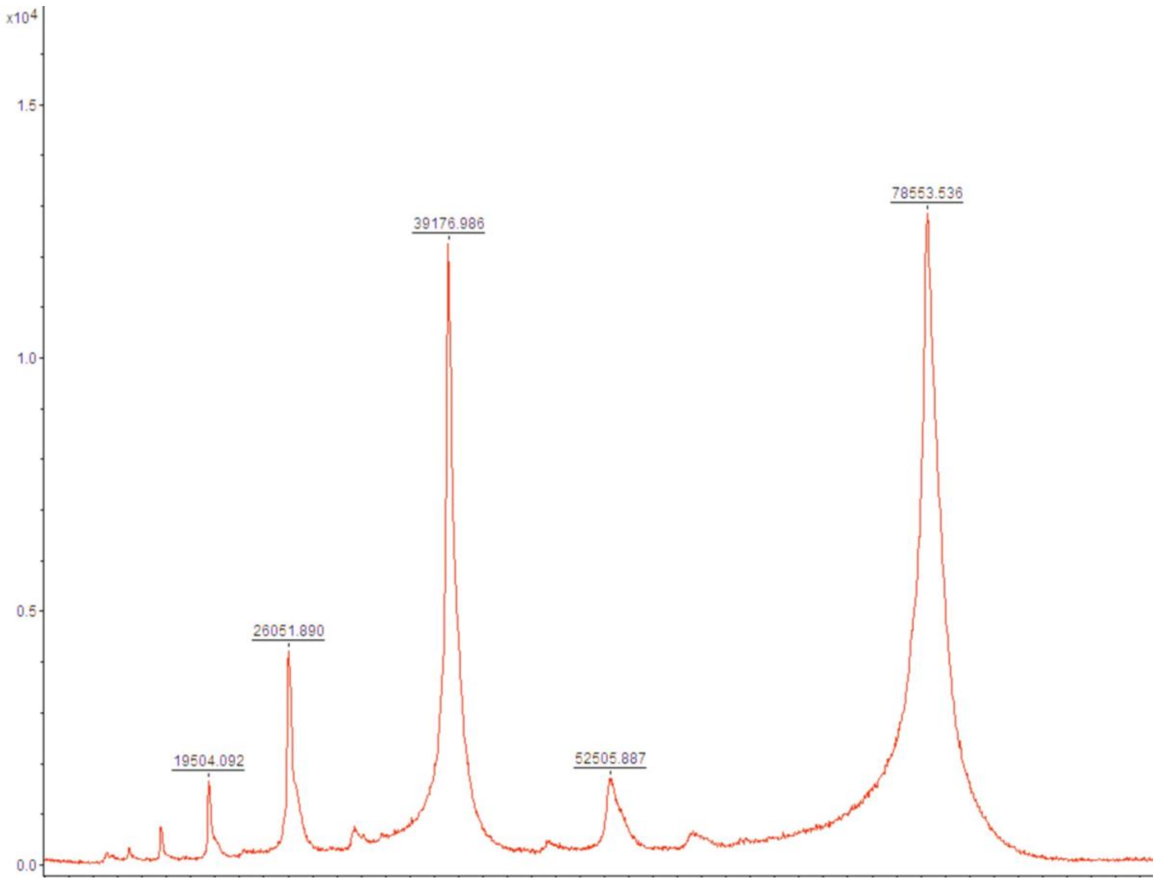

Figure S4. MALDI-TOF analysis of purified β-galactosidase: β-galactosidase shows two major peaks around 78.55 kDa (calculated size: 79147 including 7His) and 39.176 kDa (dimer).

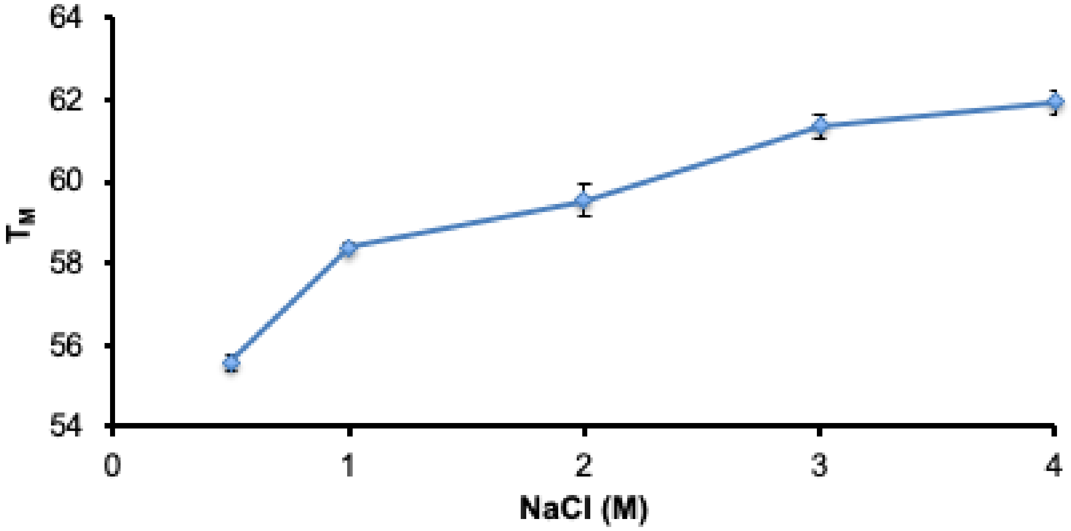

Figure S5. Measured melting temperatures at different salt concentrations

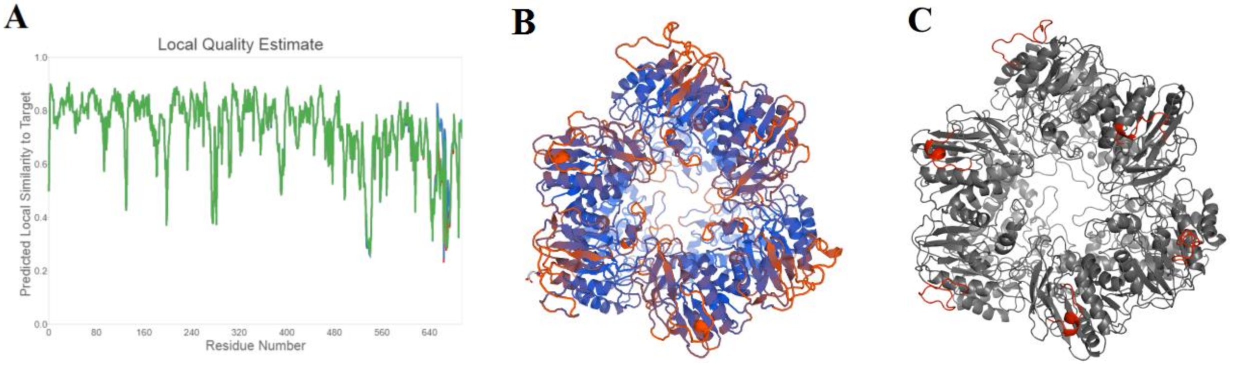

Figure S6. Structural assessment of the hla_bga homology model produced by SwissModeller [16]. (A) Local quality estimates are shown for each amino acid position of the monomer chain A. The overall global score of the homology model is 0.73 ± 0.05, global score ranges between 0 to 1 method used was QMEANDisco [16]. (B) Local quality scores shown on the structure model, red color indicates poor score and blue color indicates good score. Pictures were derived from the server. (C) Modelled hla_bga, the modelled loop regions not visible in the electron density are shown in magenta color.

T-Coffee

```
Hla_bga             MRLGVCYFPEHWPSE--EWERDVAAMADAGLEYVRMAEFSWGVLEPERGT
1KWG                M-LGVCYYPEHWPKE--RWKEDARRMREAGLSHVRIGEFAWALLEPEPGR
4UZS                LWYGGDYNPDQWPE--EVWDDDIRLMKKAGVNLVSVGIFSWAKIEPEEGK
3TTS                IWYGGDYNPEQWDK--ATMEEDMRMFNLAGIDVATVNVFSWAKIQRDEVS
4UNI                IWFGADYNPDQWPE--DVQDEDIRLMKQAGVNIVSLAIFSWANIETSDGN
5XB7                ILFGAAYYDEYIPRDLDRIDTDMEMMTRAGINVIRIGESTWSTCEPQPGH
4OIF                ILHGGDYNPDQWLDRPDILQADLELMKLSHTNTFTVGVFAWSALEPEEGV
5E9A                LLHGADYNPEQWLDHPDVLVRDVEMMKEARCNVMSVGIFSWSALEPEEGR
6Y2K                MKLGVCYYPEHWPKS--RWVEDAQHMRRIGIQYVRVGEFSWSTIEPTPGE
                    :  *  *  :           *   :     .   :   :*.  :

Hla_bga             FDFEWLDEAIELIGDHGMQAVLCTPTATPPKWLVDERPSIRQEDPDGTVR
1KWG                LEWGWLDEAIATLAAEGLKVVLGTPTATPPKWLVDRYPEILPVDREGRRR
4UZS                YDFDWLDRAIDKLGKAGIAVDLASATASPPMWLTQAHPEVLWKDERGDTV
3TTS                YDFTWLDDIIERLTKENIYLCLATSTGAHPAWMAKKYPDVLRVDYEGRKR
4UNI                FEFDWLDRVIDKLYKAGIAVDLASATASPPMWLTSAHPEVLRRDEQGHVI
5XB7                FDWTHIDRALDAATNAGINVIVGTPTYAVPTWLVAMYPDVLATTPAGEP-
4OIF                YRFEWLDKVFDDIYRIGGRVILATPSGARPAWLSQKYPEVLRVNAARVRQ
5E9A                YTFDWMDQVLNRLHENGISVFLATPSGARPAWMSQKYPQVLRVGRDRVPA
6Y2K                LHWEWLDESLDILHSQGLKVILGTPTATPPKWLVDRHPSMLAKDEAGRVR
                      :  :*  :      .    : :.: : * *:    *.:

Hla_bga             EHGSRRHYCFNSDAYREETARIVERVTERYADSPHVAGWQTDNEFGCHET
1KWG                RFGGRRHYCFSSPVYREEARRIVTLLAERYGGLEAVAGFQTDNEYGCHDT
4UZS                WPGAREHWRPTSPVFREYALNLCRRMAEHYKGNPYVVAWHVSNEYGCHNR
3TTS                KFGGRHNSCPNSPTYRKYAKILAGKLAERYKDHPQIVMWHVSNEYGGY--
4UNI                WPGARQHWRPTSPTFRTYALRLCREMAEHYKDNPAIVSWHVGNEYGCHNY
5XB7                HYGARQIMNIVNPAYRLYGERVIRSLISHVAQQPCVIGYQVDNETKYYDS
4OIF                LHGGRHNHCFTSSVYREKTQHINRLLAERYGDHPALLMWHVSNEYGGE--
5E9A                LHGGRHNHCMSSPVYREKVQLMNGQLAKRYAHHPAVIGWHISNEYGGE--
6Y2K                GFGSRRHYTFASLEYREECRRMVTMMAERYGHHPAVASWQTDNEYGCHDT
                      *.*.     .  :*     :   : .:      :  :: .**

Hla_bga             VRCYCDDCADAFRTWLADRY-G-DIDRLNEAWGNAFWSQQYGSFDEIDPP
1KWG                VRCYCPRCQEAFRGWLEARY-G-TIEALNEAWGTAFWSQRYRSFAEVELP
4UZS                -FDYSDDAMRAFQKWCKKRY-K-TIDAVNEAWGTAFWAQHMNDFSEIIPP
3TTS                --CYCDNCEKQFRVWLKERY-G-TLEALNKAWNTSFWSHTFYDWDEIVAP
4UNI                -FDYSDDAVQAFREWCRDRY-G-TIDKVNAAWGTNFWSQRLNSFEEILPP
5XB7                V---SHDMQVMFIKQLRHEFKN-DLEALNEAYGLDYWSNRINAWEDF--P
4OIF                --CHCNLCQEAFREWLKKKY-NHDLDALNAAWWTSFWSHTYTDWSQIESP
5E9A                --CHCDTCQGQFRDWLKARY-V-TLDALNKAWWSTFWSHTYTDWSQLESP
6Y2K                VLSYAEADLAAFRLWLAEKY-G-TVEALNKAWGNVFWSMDYRSFDEIELP
                        .      *      .:    :: :* *:   :*:     : :.  *

Hla_bga             GPTPAEH------HPSRLLAYARFSSDSVVEYNRLHADLIREADP-DWFV
1KWG                HLTVAEP------NPSHLLDYYRFASDQVRAFNRLQVEILRAHAP-GKFV
4UZS                RYIGD-GN--F-MNPGKLLDYKRFSSDALKELYIAERDVLESITP-GLPL
3TTS                NALSEEWSGNRTNFQGISLDYRRFQSDSLLECFKMERDELKRWTP-DIPV
4UNI                RYVGGEGN--F-TNPGRLLDFKHFCSDALKEFFCAERDVLSEVTP-NIPL
5XB7                DLTGS-I------NESLRARFDRFRRDQVAEYLAWQASIIREYMRDDQFI
4OIF                SPIGEHT------IHGLNLDWKRFVTDQTISFFENEIVPLRELTP-HIPI
5E9A                SPQGENG------VHGLNLDWRRFNTDQVTRFCSEEIRPLKAENP-ALPA
6Y2K                NLTVTEA------NPSHRLDFQRCCSDQVVAFNKLQVDILREHSA-GRDL
                                   .    : :   *        .   :
```

```
Hla_bga         THNFM--------GR------FPTLNAYDVSEDLDRVAWDSYPTGFVQDR
1KWG            THNFM--------GF------FTDLDAFALAQDLDFASWDSYPLGFTDLM
4UZS            TTNFM--------VSA----GGSMLDYDDWGAEVDFVSNDHYFTPGEA--
3TTS            TTNLM--------GF------YPELDYFKWAKEMDVVSWDNYPSMDTP--
4UNI            TTNFM--------VSA----SQNTLDYDDWAHEVDFVSNDHYFTPGSW--
5XB7            THNFDYEWRGHSYGL------QPAVDHFRAARALDICGVDIYHPSEDA--
4OIF            TTNFM--------ADTHDLIPFQGLDYSKFAKHLDVISWDAYPAWHNDWE
5E9A            TTNFM--------EY------FNDYDYWKLAGVLDFISWDSYPMWHTRQD
6Y2K            VHNYM--------GF------FTAFDHHKVGQDLDVASWDSYPLGSLDKE
                . *                       :    .  :*  . * *

Hla_bga         -Y-DGEASPDQLRAGDPDQVGMDHDIYRSALDR-PFWVMEQQ-PGDVNWP
1KWG            PL-PPEEKLRYARTGHPDVAAFHHDLYRGVGRG-RFWVMEQQ-PGPVNWA
4UZS            ---------------HFDEVAYAASLMDGISRKEPWFQMEHS-TSAVNWR
3TTS            ----------------FSFTAMAHNLMRGLKSGQPFMLMEQT-PGVQNWQ
4UNI            ---------------HIDELAYSASLVDGISRKKPWFLMEQS-TSAVNWR
5XB7            --------------LTGKEIAFGGDMARSAGGG-NYLVLETQAQGQHGWL
4OIF            ST-----------ADLAMKVGFINDLYRSLKQQ-PFLLMECT-PSLVNWH
5E9A            DI------------GLAAYTAMYHDLMRTLKQGKPFVLMEST-PSFTNWQ
6Y2K            PLYTEDEKHTYLRVGHPDAGAFHHDLYRGCGNG-RLWIMEQQ-PGPVNWA
                                    .   .:            :*    .  .*

Hla_bga         PHCPQPGEGAMRLWAHHAAAHGADAVLYFRWRRCLEGQEQYHAGLRKADG
1KWG            PHNPSPAPGMVRLWTWEALAHGAEVVSYFRWRQAPFAQEQMHAGLHRPDS
4UZS            PINYRAEPGSVVRDSLAQVAMGADAICYFQWRQSKAGAEKWHSSMVPHAG
3TTS            PYNSAKRPGVMRLWSYQAVAHGADTVMFFQLRRSVGACEKYHGAVIEHVG
4UNI            EINPRKEPGELIRDSMLHLAMGADAICYFQWRQSRSGAEKFHSAMLPLAG
5XB7            P-----YPGQLRLQAYSHLASGADGIMYWHWHSIHNSFETYWRGLLSHDF
4OIF            KVNKAKRPGMHFLSSMQMIAHGSDSILYFQWRKSRGSFEKFHGAVVDHDN
5E9A            PTSKLKKPGMHILSSLQAVAHGADSVQYFQWRKSRGSCEKFHGAVVDHVG
6Y2K            PHNPTPADGAVRLWTWEAFSHGAELVSYFRWRQAPFGQEQMHAGLLRPDA
                        *     :    : *:: : ::: :    . *    .:

Hla_bga         S-PDRGYADAAHTSEEFATLD----------GASHVDAPVAVVFDYDSLW
1KWG            A-PDQGFFEAKRVAEELAALA----------LPPVAQAPVALVFDYEAAW
4UZS            E-DSQIFRDVCELGADLGRLSDE-----GLMGTKTVKSKVAVVFDYESQW
3TTS            HEHTRVFRECAELGKELQQLGDT------ILD-ARSEAKVAVMYDWENRW
4UNI            E-HSQIYRDVCALGADLDTLSDA-----GILRSKLSKARVAIVQDIQSEW
5XB7            E-SNPTYEEAGRFGREIGDPR-----IGDTLSHLSKRNAVAILASNESLT
4OIF            RTDSRVFQEVAEVGKALKKMSGI-------VG-TNRPAEVAILYDWENNW
5E9A            HIDTRVGREVAELGSILSALAPV-------AG-SRVEAKVAIIFDWESRW
6Y2K            Q-EAEAAKEATLVAQEVKVLAESIGLDADELMSLPSAGKVALMFDYDACW
                        :    .  .                      **:: . :

Hla_bga         ALN----A--QPHAPDFDYWALQEAFYGAVRGRGVQVDVVPPSAD---LS
1KWG            IYE----V--QPQGAEWSYLGLVYLFYSALRRLGLDVDVVPPGAS---LR
4UZS            ATE----YTANPTQ-QVDHWTEPLDWFRALADNGITADVVPVRSD---WD
3TTS            ALE----LSSGPSI-ALNYVNEVHKYYDALYKQNIQTDMISVEED---LS
4UNI            ATE----HTATPTQ-HIREWTEPLDWFAAFANRGVTADVTPIHAQ---WD
5XB7            ALSWFHIETGFPMGGTLTYNDVLRSIYDALFELNVEVDFLPADASADQLA
4OIF            ALN----DAQGFAAETKRYPQTLVQHYRPFWERDIPVDVITKEHD---FS
5E9A            AMD----DAMGPRNAGLHYENTVADHYRALWAQGIAVDVINADCD---LQ
6Y2K            SLD----I--QPQSRAYRYFFWCYRMYEAMRELGLSVDIVPSNAP---LD
                  .                       : ..   .: .*.
```

```
Hla_bga         GYAAVVAPALHLVTEDLADRLTDYIAGGGEVLFGPRTGVKDAENKLRPMS
1KWG            GYAFAVVPSLPIVREEALEAFREA---EGPVLFGPRSGSKTETFQIPKEL
4UZS            SYEIAVLPCVYLLSEETSRRVREFVANGGKLFVTYYTGLSDENDHIWLGG
3TTS            KYKVVIAPVMYMVKPGFAERVERFVAQGGTFVTTFFSGIVNENDLVTLGG
4UNI            TYDAVVIPCVYLFSEEMAERLRTFVRNGGKAFVTYYSALADEHDRLHTEG
5XB7            GYSLVIAPALYTTDQQTIDRLARYVKNGGHLLATMRSFVADENVKVWHDK
4OIF            RYKLLIAPMLYLVSEETIARLKEFVANGGTLVMTYISGIVDEHDLAYLGG
5E9A            GYDLVIAPMLYMVREGVGERISAFVQAGGRFVATYWSGIVNETDLCFLNG
6Y2K            MYELLVLPAQAHITPELQNRLNSY---QGVLLAGPRTGSKTETYQIPENL
                 *    : *               .         *  .     :

Hla_bga         QPGPLTDLVGATVDQHES-L---PRRLE--TTVRRVGDPTDDSEEIAAPP
1KWG            PPGPLQALLPLKVVRVES-L---PPGLL--EVAEGA-----------LGR
4UZS            YPGSIRDVVGVRVEEFAP-MGNDMPGAL--DHLDLD------------NG
3TTS            YPGELRNVMGIWAEEIDA-L---LPGHQ--NEIVLRQ----DWGGLR-GS
4UNI            WPGLIGDVVGVRIEEHCP-LGTLFPGML--DHLDVS------------NG
5XB7            APHHLVDIFGMTYNQFTRPM---GVSLKCPDTLADL-----------AGA
4OIF            WHQDLREMFGMEPIETDT-L---YPRDR--NSVHYRG-----------RS
5E9A            FPGPLRPVLGIWAEEIDS-L---TDEQH--NSVAGVE---GNALGLS-GP
6Y2K            APGPLASLLPLTVERVDA-L---PEHTQ--PAVSGR-----------WGA
                     :  :.       .     :

Hla_bga         VSFRTWAEWLD--PDAAEPQYAYDVDGP--ADGRPAVVTNTVGDGQVTYC
1KWG            FPLGLWREWVE--APLKPL-LTF-------QDGKGA----LYREGRYLYL
4UZS            TVAHDFADVITSTADTSTVLASYKAERWTGMNEVPAIVANGYGDGRTVYV
3TTS            YSCGILCDVIH--AETAEVLAEYGADYY---KGTPVLTRNKFGNGQSYYV
4UNI            TVVHDLADVIDAIADDTTVLATFEADPATGMDGRAAITVHPYHEGGVAYI
5XB7            -SANDFIEMLSP-APETHVLAWYDHYA---WDSYAAITRHAFGSGDAQWV
4OIF            YELKDYATVIK--IHAATVEGVYEDDFY---ADTPAVTSNQYGKGQAYYI
5E9A            YRASQLCEVIH--LEGAAALATYGDDFY---AGNPAVTVNLYGKGQAYYV
6Y2K            GKLKHWHEQIK--TELPCL-LKD-------DGGNPV----LMGEGRHYYL
                           :                              .         .*    :

Hla_bga         GVWPESDLADALASDLLDRAGVRYAERL--PDGVRIGY--R---GGRTWV
1KWG            AAWPSPELAGRLLSALAAEAGLKVL-SL--PEGLRLRR--R---GTWVFA
4UZS            GCRLGRQGLAKSLPAMLGSMGLSDLAG--DGRVLRVERADAAAASHFEFV
3TTS            ASSPDADFLQGLIANLCEEQGVKPLL--NTPDGVEVAER-VKNGTSYLFV
4UNI            AGKLGRDGISQSLPEICAALGFELDADPRAGDVLRVVREQ-EDGAIFEFL
5XB7            GTQLQADAWRTVLAEALSNAGVHTP-GMELAGTVCVRSGTNTAGDTVTYL
4OIF            GGRLEDQFHRDFYQELMEKLDLRPVLFVKHEKGVSVQAR-QAPECDYVFI
5E9A            ASRNDQQFHADFFTALAKEMKLPRAINTPLPEGVTAARR-TDGESEFIFL
6Y2K            GSCIDNTLLKASLAKLSEVAGLSTY-YL--PKGVRVRE--R---GNVIFA
                .                        .              :                 :

Hla_bga         TNFTSDRLRLPEIDPESLAVDDTDRDGFDPMADD-DK--DSSAD------
1KWG            FNYGPEAVEAPA--------------------SE-GA--RFLLG------
4UZS            FNRTHEPVTVDV--------------------EG-EA-IAASLAH-----
3TTS            MNHNAEEMTFDA--------------------GASRQR-DLLTGK-----
4UNI            FNRTRNTVTADR--------------------PA-GDMLICSLAT-----
5XB7            LNYSGSPITFRA--------------------PA-SG--TFLLGHPTDDG
4OIF            MNFTEEKQAVVL--------------------EE-KVK-DLFTGE-----
5E9A            QNYNADNQTVAL--------------------PQ-DYQ-DIVHGG-----
6Y2K            FNYSSNTVVFEP----------------------Q-NA--ELVIG------
                 *    .
```

```
Hla_bga         ---------------GIVVGPYGVAVIEGDCVD
1KWG            ---------------SRRVGPYDLAVW--E---
4UZS            ----------VDDGRATIDPTGVVVLR----R
3TTS            ----------TISGQATIPARGVMILER---A
4UNI            ----------DSTDKVTLEPNGVLAF-----R
5XB7            EQAVTAETPVTVGDAVTLPRWGVDIIVGRQPT
4OIF            ----------EIVGEIMLDKYEVRVVEKR--R
5E9A            ----------NLPRKLTLPAFGCQILTR---K
6Y2K            ---------------SMCLGAADVAIWKKQ---
                                 :
```

Figure S7. Sequence alignment obtained with T-Coffee [17]. The red blocks represent the β-galactose binding site, predicted flexible loops of hla_bga (532-543 and 657-672) are highlighted in yellow shade. *Halorubrum lacusprofundi*, hla; *Thermus thermophilus* A4, 1KWG; *Bifidobacterium bifidum* S17, 4UZS; *Bacillus circulans* sp. Alkalophilus, 3TTS; *Bifidobacterium animalis* subsp. lactis Bl-04, 4UNI; *Bifidobacterium* species 5XB7; *Geobacillus stearothermophilus*, 4OIF; *Rahnella* sp. R3, 5E9A; *Marinomonas* ef1, 6Y2K

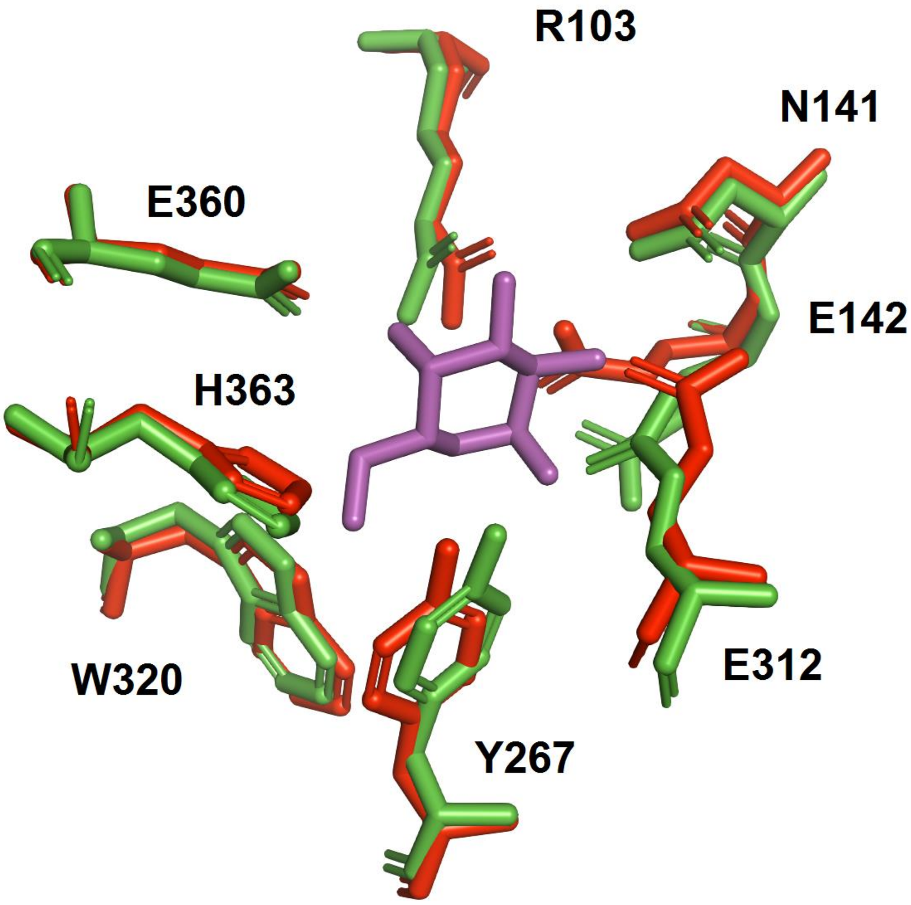

Figure S8. β-D-galactose binding pocket. The binding pocket of hla_bga based on conserved catalytic motif of various β-Gal structures. The amino acids residues of hla_bga, β-Gal-tA4 (1KWK) and β-D-galactose are red, green and magenta respectively. 1KWK and 1KWG are β-Gal-tA4 with β-D-galactose ligand and without β-D-galactose ligand, respectively.

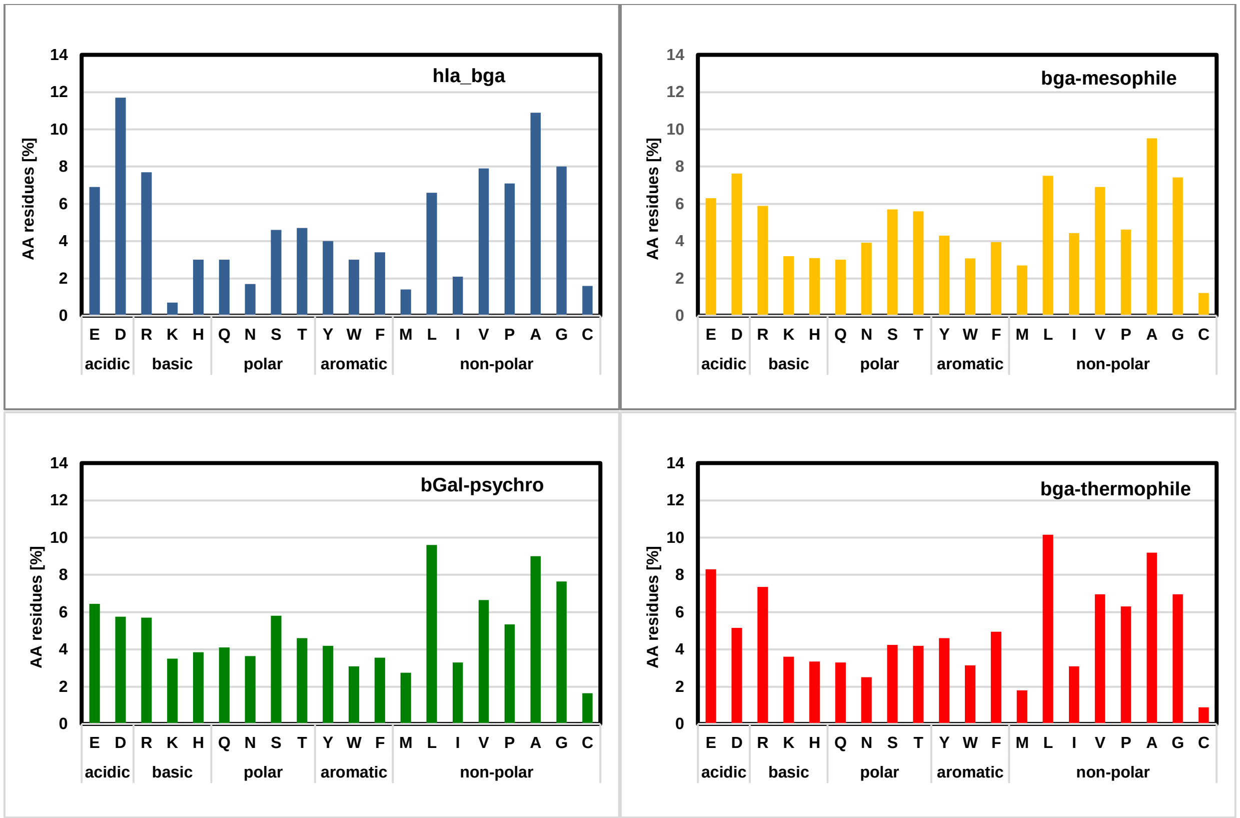

Figure S9. Ratio of amino acids in hla_bga and its structural homologs. *Halorubrum lacusprofundi*, hla_bga; bga-mesophile (3TTS, 4UZS, 4UNI, 5XB7); bga-psychrophile (5E9A, 6Y2K); bga-thermophile (1KWG, 4OIF).

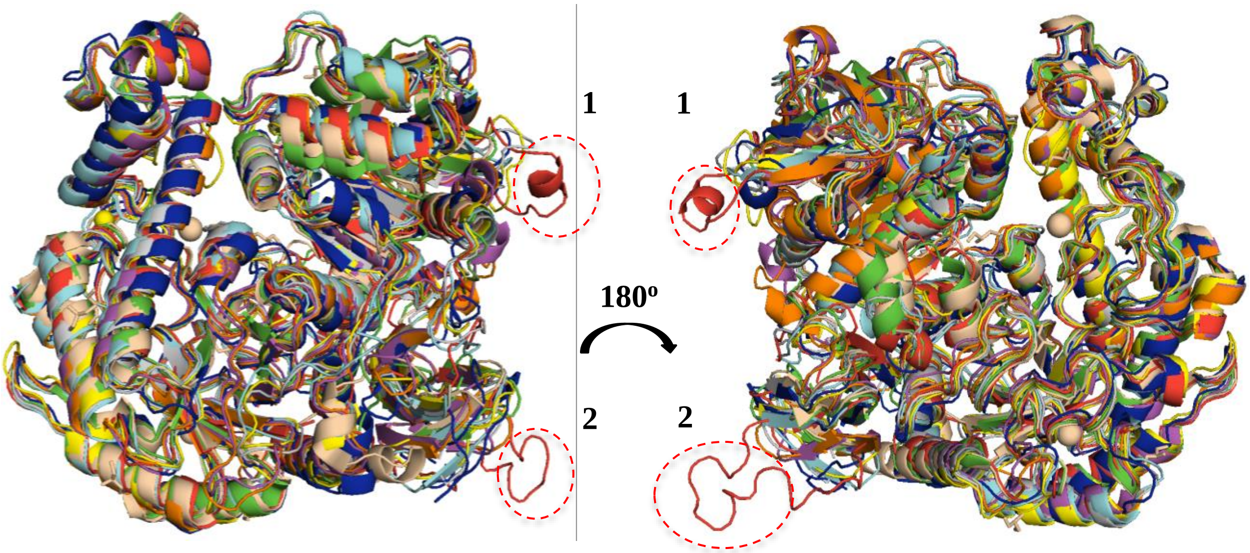

Figure S10. Superimposed β-Galactosidase monomers: 1 and 2 represent the flexible loop regions 532-543 and 657-672, respectively, of the modeled bga_hla. *Halorubrum lacusprofundi*, hla (red); *Thermus thermophilus* A4, 1KWG (green); *Bacillus circulans* sp. *Alkalophilus*, 3TTS (yellow); *Bifidobacterium animalis* subsp. lactis Bl-04, 4UNI (orange); *Bifidobacterium bifidum* S17, 4UZS (pink); *Marinomonas* ef1, 6Y2K (wheat); *Rahnella* sp. R3, 5E9A (grey); *Geobacillus stearothermophilus*, 4OIF (cyan); *Bifidobacterium* species 5XB7 (blue).

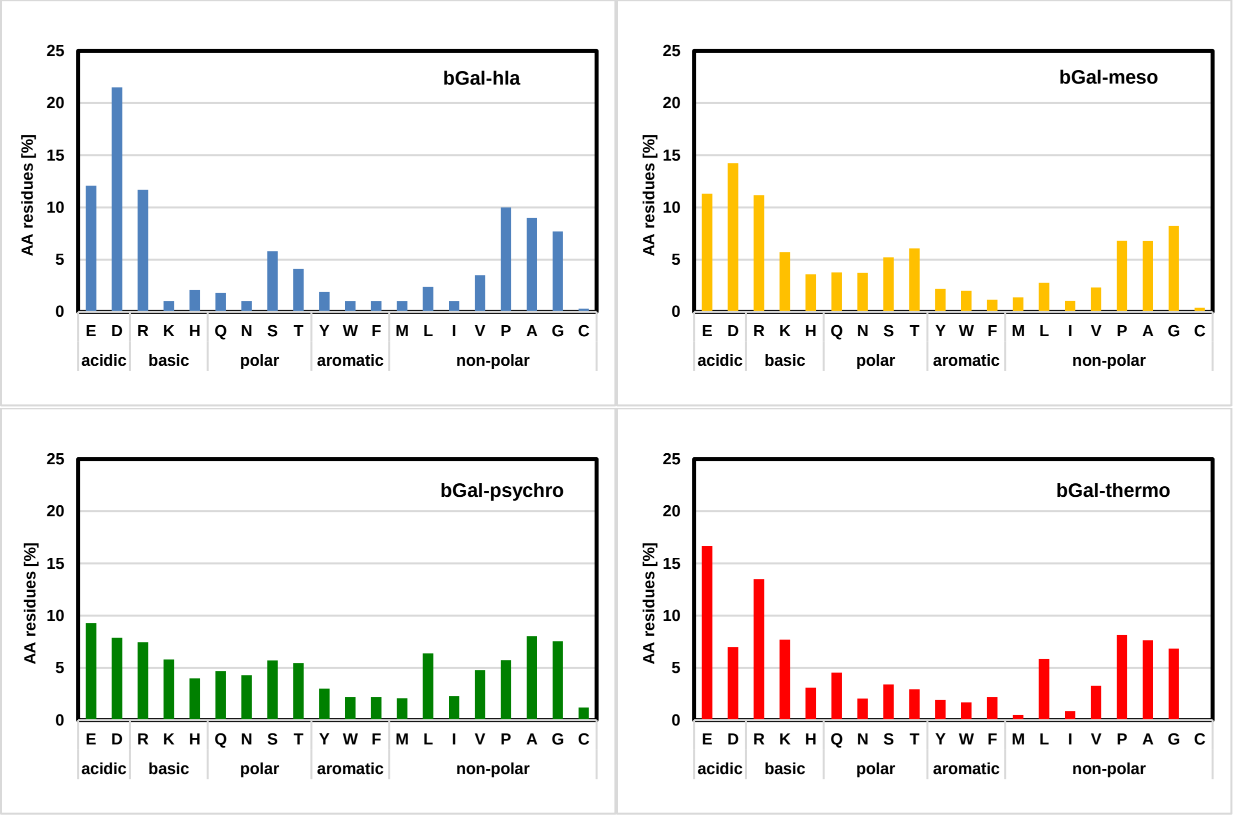

Figure S11. Ratio of amino acids on the solvent-exposed surface in hla_bga and its structural homologs. *Halorubrum lacusprofundi*, hla_bga; bga-mesophile (3TTS, 4UZS, 4UNI, 5XB7); bga-psychrophile (5E9A, 6Y2K); bga-thermophile (1KWG, 4OIF).

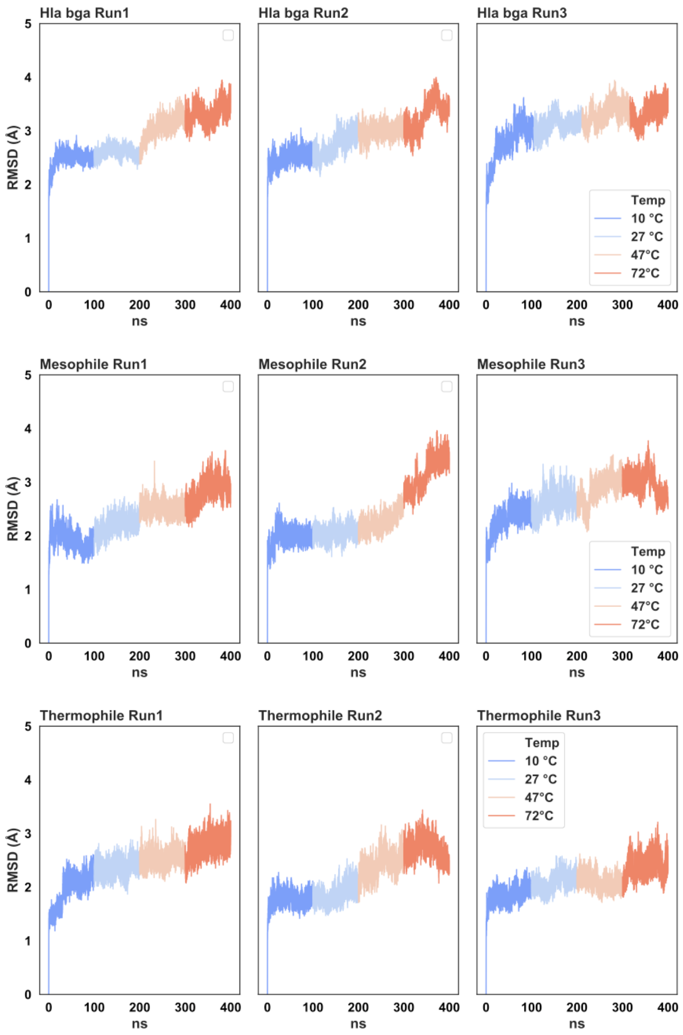

Figure S12. Backbone RMSD plots over time for the three independent simulations of the three investigated systems at the four explored temperatures (10, 27, 47 and 72 °C).

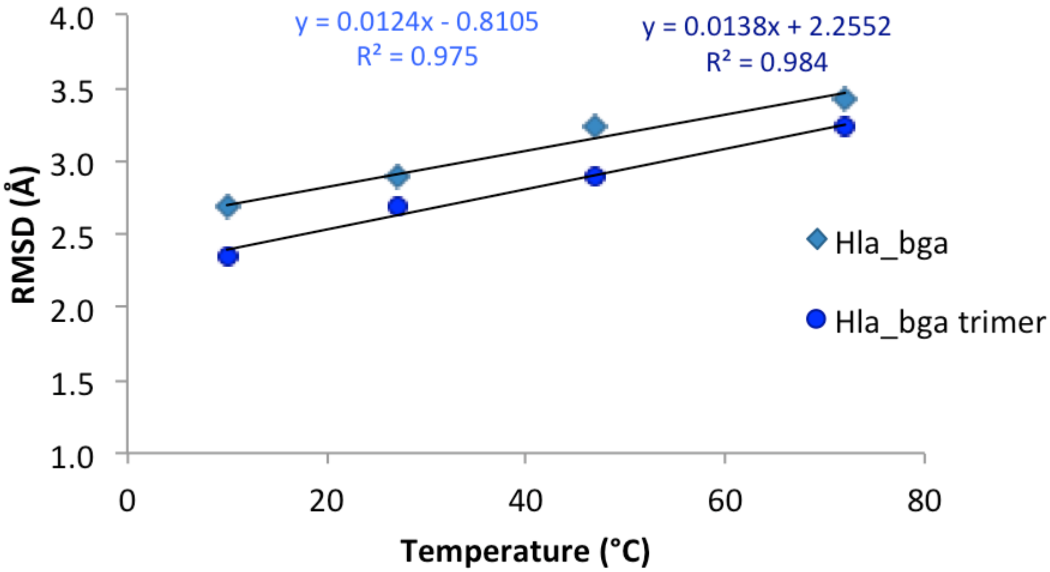

Figure S13. Effect of temperature on the hla_bga monomer and trimer backbone RMSD. Values are averaged on the last 20 ns of the simulations for each system. Corresponding trendlines are shown with relative correlation coefficients (R2) and equations.

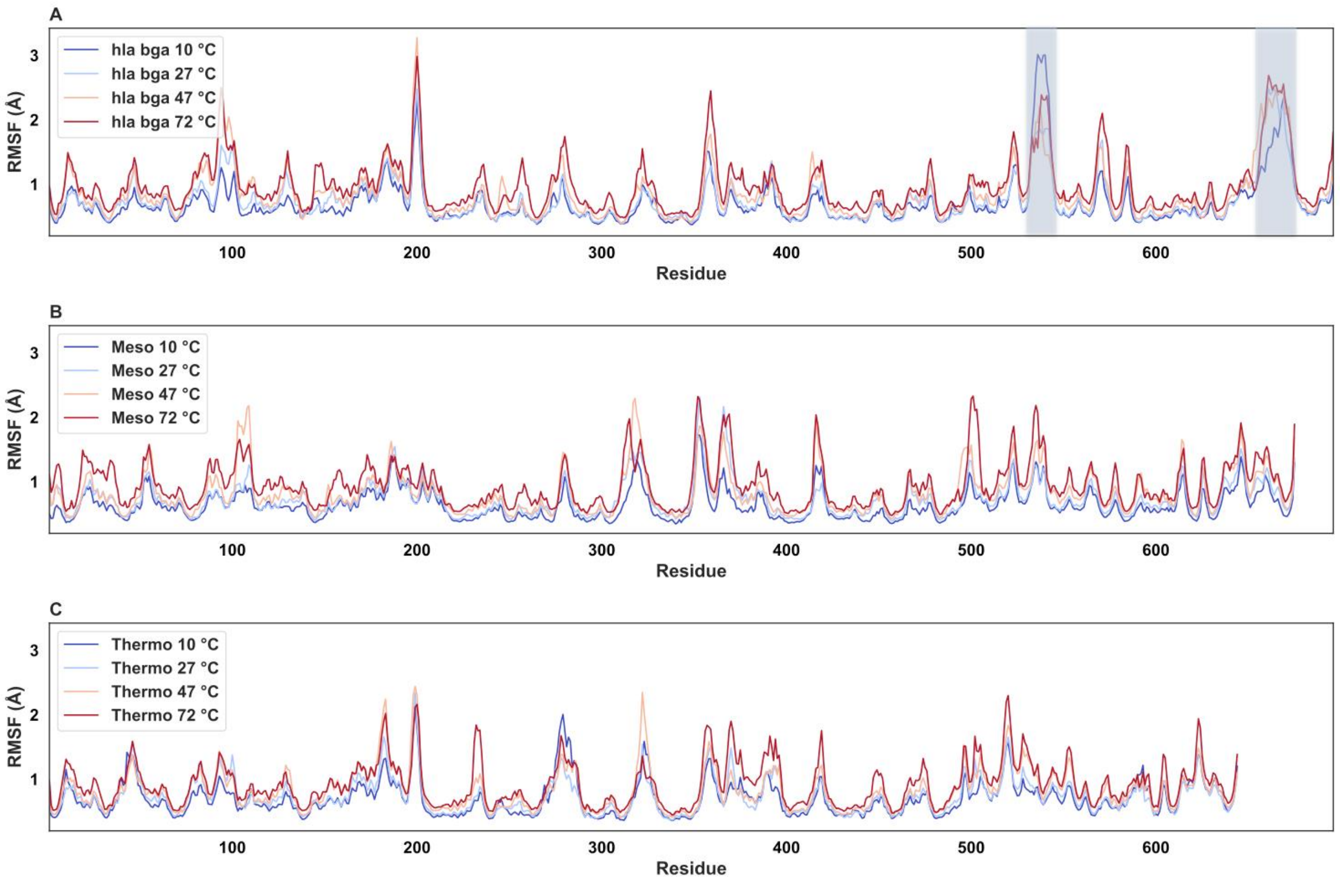

Figure S14. RMSF values averaged over the last 20 ns of the three simulations ran for each investigated system at 10, 27, 47 and 72 °C. Hla_bga loops 532-543 and 657-672 are highlighted by a blueish shadow.

References

1. DasSarma, S.; Capes, M.D.; Karan, R.; DasSarma, P. Amino acid substitutions in cold-adapted proteins from Halorubrum lacusprofundi, an extremely halophilic microbe from Antarctica. *PLoS One* **2013**, *8*, e58587.
2. Hidaka, M.; Fushinobu, S.; Ohtsu, N.; Motoshima, H.; Matsuzawa, H.; Shoun, H.; Wakagi, T. Trimeric crystal structure of the glycoside hydrolase family 42 β-galactosidase from Thermus thermophilus A4 and the structure of its complex with galactose. *Journal of molecular biology* **2002**, *322*, 79-91.
3. Ohtsu, N.; Motoshima, H.; Goto, K.; Tsukasaki, F.; MATsUzAWA, H. Thermostable β-galactosidase from an extreme thermophile, Thermus sp. A4: enzyme purification and characterization, and gene cloning and sequencing. *Bioscience, biotechnology, and biochemistry* **1998**, *62*, 1539-1545.
4. Fan, Y.; Yi, J.; Hua, X.; Feng, Y.; Yang, R.; Zhang, Y. Structure analysis of a glycosides hydrolase family 42 cold-adapted β-galactosidase from Rahnella sp. R3. *RSC advances* **2016**, *6*, 37362-37369.
5. Fan, Y.; Hua, X.; Zhang, Y.; Feng, Y.; Shen, Q.; Dong, J.; Zhao, W.; Zhang, W.; Jin, Z.; Yang, R. Cloning, expression and structural stability of a cold-adapted β-galactosidase from Rahnella sp. R3. *Protein expression and purification* **2015**, *115*, 158-164.
6. Godoy, A.S.; Camilo, C.M.; Kadowaki, M.A.; Muniz, H.d.S.; Espirito Santo, M.; Murakami, M.T.; Nascimento, A.S.; Polikarpov, I. Crystal structure of β1→6‐galactosidase from Bifidobacterium bifidum S17: trimeric architecture, molecular determinants of the enzymatic activity and its inhibition by α‐galactose. *The FEBS journal* **2016**, *283*, 4097-4112.
7. Maksimainen, M.; Paavilainen, S.; Hakulinen, N.; Rouvinen, J. Structural analysis, enzymatic characterization, and catalytic mechanisms of β‐galactosidase from Bacillus circulans sp. alkalophilus. *The FEBS journal* **2012**, *279*, 1788-1798.
8. Solomon, H.V.; Tabachnikov, O.; Lansky, S.; Salama, R.; Feinberg, H.; Shoham, Y.; Shoham, G. Structure–function relationships in Gan42B, an intracellular GH42 β-galactosidase from Geobacillus stearothermophilus. *Acta Crystallographica Section D: Biological Crystallography* **2015**, *71*, 2433-2448.
9. Tabachnikov, O.; Shoham, Y. Functional characterization of the galactan utilization system of G eobacillus stearothermophilus. *The FEBS journal* **2013**, *280*, 950-964.
10. Viborg, A.H.; Fredslund, F.; Katayama, T.; Nielsen, S.K.; Svensson, B.; Kitaoka, M.; Lo Leggio, L.; Abou Hachem, M. A β1‐6/β1‐3 galactosidase from B ifidobacterium animalis subsp. lactis B l‐04 gives insight into sub‐specificities of β‐galactoside catabolism within B ifidobacterium. *Molecular microbiology* **2014**, *94*, 1024-1040.
11. Viborg, A.H.; Katayama, T.; Arakawa, T.; Hachem, M.A.; Leggio, L.L.; Kitaoka, M.; Svensson, B.; Fushinobu, S. Discovery of α-L-arabinopyranosidases from human gut microbiome expands the diversity within glycoside hydrolase family 42. *Journal of Biological Chemistry* **2017**, *292*, 21092-21101.
12. Mangiagalli, M.; Lapi, M.; Maione, S.; Orlando, M.; Brocca, S.; Pesce, A.; Barbiroli, A.; Camilloni, C.; Pucciarelli, S.; Lotti, M., et al. The co-existence of cold activity and

thermal stability in an Antarctic GH42 β-galactosidase relies on its hexameric quaternary arrangement. *The FEBS Journal n/a*, doi:10.1111/febs.15354.

13. Graziano, G.; Merlino, A. Molecular bases of protein halotolerance. *Biochim Biophys Acta* **2014**, *1844*, 850-858, doi:10.1016/j.bbapap.2014.02.018.
14. Santiago, M.; Ramírez-Sarmiento, C.A.; Zamora, R.A.; Parra, L.P.J.F.i.m. Discovery, molecular mechanisms, and industrial applications of cold-active enzymes. **2016**, *7*, 1408.
15. Karshikoff, A.; Ladenstein, R.J.P.e. Proteins from thermophilic and mesophilic organisms essentially do not differ in packing. **1998**, *11*, 867-872.
16. Waterhouse, A.; Bertoni, M.; Bienert, S.; Studer, G.; Tauriello, G.; Gumienny, R.; Heer, F.T.; de Beer, T.A P.; Rempfer, C.; Bordoli, L., et al. SWISS-MODEL: homology modelling of protein structures and complexes. *Nucleic Acids Research* **2018**, *46*, W296-W303, doi:10.1093/nar/gky427.
17. Zimmermann, L.; Stephens, A.; Nam, S.Z.; Rau, D.; Kübler, J.; Lozajic, M.; Gabler, F.; Söding, J.; Lupas, A.N.; Alva, V. A Completely Reimplemented MPI Bioinformatics Toolkit with a New HHpred Server at its Core. *J Mol Biol* **2018**, *430*, 2237-2243, doi:10.1016/j.jmb.2017.12.007.
